# Alcohol-related breast cancer in postmenopausal women – effect of *CYP19A1*, *PPARG* and *PPARGC1A* polymorphisms on female sex-hormone levels and interaction with alcohol consumption and NSAID usage in a nested case-control study and a randomised controlled trial

**DOI:** 10.1186/s12885-016-2317-y

**Published:** 2016-04-21

**Authors:** Tine Iskov Kopp, Ditte Marie Jensen, Gitte Ravn-Haren, Arieh Cohen, Helle Molgaard Sommer, Lars Ove Dragsted, Anne Tjonneland, David Michael Hougaard, Ulla Vogel

**Affiliations:** Technical University of Denmark, National Food Institute, Søborg, Denmark; Danish Cancer Society Research Center, Copenhagen, Denmark; Research Centre for Prevention and Health, Rigshospitalet - Glostrup, Glostrup, Denmark; Section of Environmental Health, University of Copenhagen, Copenhagen, Denmark; Department of Clinical Biochemistry and Immunology, Statens Serum Institute, Copenhagen, Denmark; Department of Nutrition, Exercise and Sports, University of Copenhagen, Copenhagen, Denmark; National Research Centre for the Working Environment, Copenhagen, Denmark

**Keywords:** Alcohol consumption, Breast cancer, Polymorphisms, *CYP19A1*, *PPARG*, Female sex-hormones, NSAID, Prospective nested case-control study, Epidemiology, Randomised controlled trial

## Abstract

**Background:**

Alcohol consumption is associated with increased risk of breast cancer (BC), and the underlying mechanism is thought to be sex-hormone driven. In vitro and observational studies suggest a mechanism involving peroxisome proliferator-activated receptor gamma (PPARγ) in a complex with peroxisome proliferator-activated receptor gamma coactivator 1-α (PGC-1α) and interaction with aromatase (encoded by *CYP19A1*). Use of non-steroidal anti-inflammatory drugs (NSAID) may also affect circulating sex-hormone levels by modifying PPARγ activity.

**Methods:**

In the present study we assessed whether genetic variation in *CYP19A1* is associated with risk of BC in a case-control study group nested within the Danish “Diet, Cancer and Health” cohort (n_cases_ = 687 and n_controls_ = 687) and searched for gene-gene interaction between *CYP19A1* and *PPARGC1A*, and *CYP19A1* and PPARG, and gene-alcohol and gene-NSAID interactions. Association between the *CYP19A1* polymorphisms and hormone levels was also examined among 339 non-HRT users. Incidence rate ratios were calculated based on Cox’ proportional hazards model. Furthermore, we performed a pilot randomised controlled trial to determine the effect of the *PPARG* Pro^12^Ala polymorphism and the PPARγ stimulator Ibuprofen on sex-hormone levels following alcohol intake in postmenopausal women (*n* = 25) using linear regression.

**Results:**

Genetic variations in *CYP19A1* were associated with hormone levels (estrone: *P*_rs11070844_ = 0.009, estrone sulphate: *P*_rs11070844_ = 0.01, *P*_rs749292_ = 0.004, *P*_rs1062033_ = 0.007 and *P*_rs10519297_ = 0.03, and sex hormone-binding globulin (SHBG): *P*_rs3751591_ = 0.03) and interacted with alcohol intake in relation to hormone levels (estrone sulphate: *P*_interaction/rs2008691_ = 0.02 and *P*_interaction/rs1062033=_ 0.03, and SHBG: *P*_interaction/rs11070844_ = 0.03). *CYP19A1*/rs3751591 was both associated with SHBG levels (*P* = 0.03) and with risk of BC (Incidence Rate Ratio = 2.12; 95 % Confidence Interval: 1.02–4.43) such that homozygous variant allele carriers had increased levels of serum SHBG and were at increased risk of BC. Acute intake of alcohol decreased blood estrone (*P* = <0.0001), estrone sulphate (*P* = <0.0001), and SHBG (*P* = 0.009) levels, whereas Ibuprofen intake and *PPARG* Pro^12^Ala genotype had no effect on hormone levels.

**Conclusions:**

Our results suggest that genetically determined variation in *CYP19A1* is associated with differences in sex hormone levels. However, the genetically determined differences in sex hormone levels were not convincingly associated with BC risk. The results therefore indicate that the genetically determined variation in *CYP19A1* contributes little to BC risk and to alcohol-mediated BC risk.

**Trial registration:**

NCT02463383, June 3, 2015.

**Electronic supplementary material:**

The online version of this article (doi:10.1186/s12885-016-2317-y) contains supplementary material, which is available to authorized users.

## Background

Alcohol is a well-known risk factor for breast cancer (BC) [1–3], and observational studies have shown that intake of alcohol is associated with 7-10 % increased risk of BC per 10 g alcohol consumed per day (defined as a unit of alcohol by the World Health Organisation) [4–9]. It is believed that at least part of the underlying mechanism is sex-hormone driven [1, 10, 11]. Several controlled experimental and observational human studies demonstrate associations between alcohol intake and increased female sex-hormone blood concentrations in pre- and postmenopausal women [12–25]. Additionally, alcohol is more strongly associated to hormone-sensitive BCs than hormone-insensitive subtypes [26]. Increased aromatization [27, 28], impairment of estrogen metabolism in the liver [27] or stimulation of adrenal steroidogenesis [17] are possible mechanisms by which alcohol increases sex-hormone concentrations in women.

Genetic epidemiology in BC research may be used to elucidate the involved molecular pathways and define subpopulations of women being more susceptible to alcohol-related BC risk. Indeed, in the Danish prospective cohort study “Diet, Cancer and Health” (DCH), variant allele carriers of the *PPARG2* Pro^12^Ala (rs1801282) polymorphism had a 20 % increased risk of BC per 10 g of alcohol consumed per day, whereas carriage of the wild type allele was not associated with alcohol-related BC [29], thus implicating peroxisome proliferator-activated receptor gamma (PPARγ) in alcohol-related breast carcinogenesis. In an updated study including 798 BC cases, the risk estimate was 13 % increased risk per 10 g alcohol per day among variant allele carriers [30].

PPARγ is a transcription factor which regulates adipocyte differentiation and expression of several adipocyte specific genes by binding to regulatory response elements in target genes as a heterodimer with retinoid X receptor (RXR) [31]. The Pro to Ala substitution caused by the single nucleotide polymorphism (SNP), *PPARG2* Pro^12^Ala, is only present in PPARγ2 isoform, which is primarily expressed in adipose tissue [31]. The *PPARG2* Pro^12^Ala substitution causes a 30 % reduction in target gene transcription [32]. In postmenopausal women, estrogens are primarily synthesized in adipose tissue, where aromatase (encoded by *CYP19A1*) catalyses the biosynthesis of estrogens [33]. Intake of alcohol increases aromatase expression in fat tissue in rats [34], and aromatase is negatively regulated by PPARγ at the transcriptional level [35, 36] by a mechanism involving binding of the PPARγ-RXR complex to peroxisome proliferator-activated receptor gamma coactivator 1-α (PGC-1α) [37]. An in vitro study has shown that ethanol inhibits the PPARγ-PGC-1α complex at physiologically relevant concentrations [30]. Moreover, Petersen et al. also showed that PGC-1α dependent co-activation of the PPARγ-complex is compromised for the rare Ala-variant of *PPARG* Pro^12^Ala. Thus, it was proposed that alcohol inhibits PPARγ-mediated inhibition of aromatase transcription, resulting in an alcohol and PPARγ-dependent increased aromatase transcription and increased levels of sex-hormones.

Several commonly used non-steroidal anti-inflammatory drugs (NSAIDs) including ibuprofen are PPARγ agonists [38]. Indeed, some NSAIDs are suspected to function as endocrine disruptors [39, 40]. In the DCH cohort study, interaction between use of NSAIDs and the *PPARG* Pro^12^Ala polymorphism in relation to alcohol-related risk of BC was observed [29]. NSAID use did not modify the risk of alcohol-related BC among *PPARG* Pro^12^Ala wild type carriers, however, among variant carriers, only users of NSAIDs were at risk of alcohol-related BC. Thus, the study indicated that NSAIDs activate the less active PPARγ ^12^Ala variant so that it has the same effect as the wild type PPARγ Pro^12^ transcription factor. In a meta-analysis, NSAIDs use has been associated with lowered risk of BC [41]. However, in the DCH cohort, female NSAID users, with an intake of more than 13 g alcohol per day had a 1.60 fold increased risk of BC compared to non-users of NSAIDs who consumed less than 3 g of alcohol per day [42], indicating that alcohol consumption modifies BC risk among NSAID users.

In the present study we further pursue the proposed mechanism of action of alcohol-related BC. We assess whether genetic variation in *CYP19A1* is associated with risk of BC in a case-control study group nested within the DCH cohort; and search for gene-gene interactions with *CYP19A1* and *PPARGC1A*, and *CYP19A1* and *PPARG*, and gene-alcohol and gene-NSAID interactions (interactions between *PPARG* and *PPARGC1A* were examined and published previously [30]). Association between the *CYP19A1* polymorphisms and hormone levels is also examined in a cross-sectional study based on the nested case-control study. Furthermore, we perform a pilot randomised controlled trial (RCT) to determine the effect of *PPARG* Pro^12^Ala and the PPARγ stimulator, Ibuprofen [38], on sex-hormone levels following alcohol intake in postmenopausal women.

## Methods

### DHC cohort study

#### Participants

The subjects were selected from the ongoing Danish DCH cohort study. The present study group has been described previously [8, 30]. In short, 79,729 women aged 50–64 years, born in Denmark, living in the Copenhagen or Aarhus areas and having no previous cancers at the time of invitation were invited to participate in the study between December 1993 and May 1997. A total of 29,875 women accepted the invitation, corresponding to 37 % of the invited women.

**Fig. 1:**
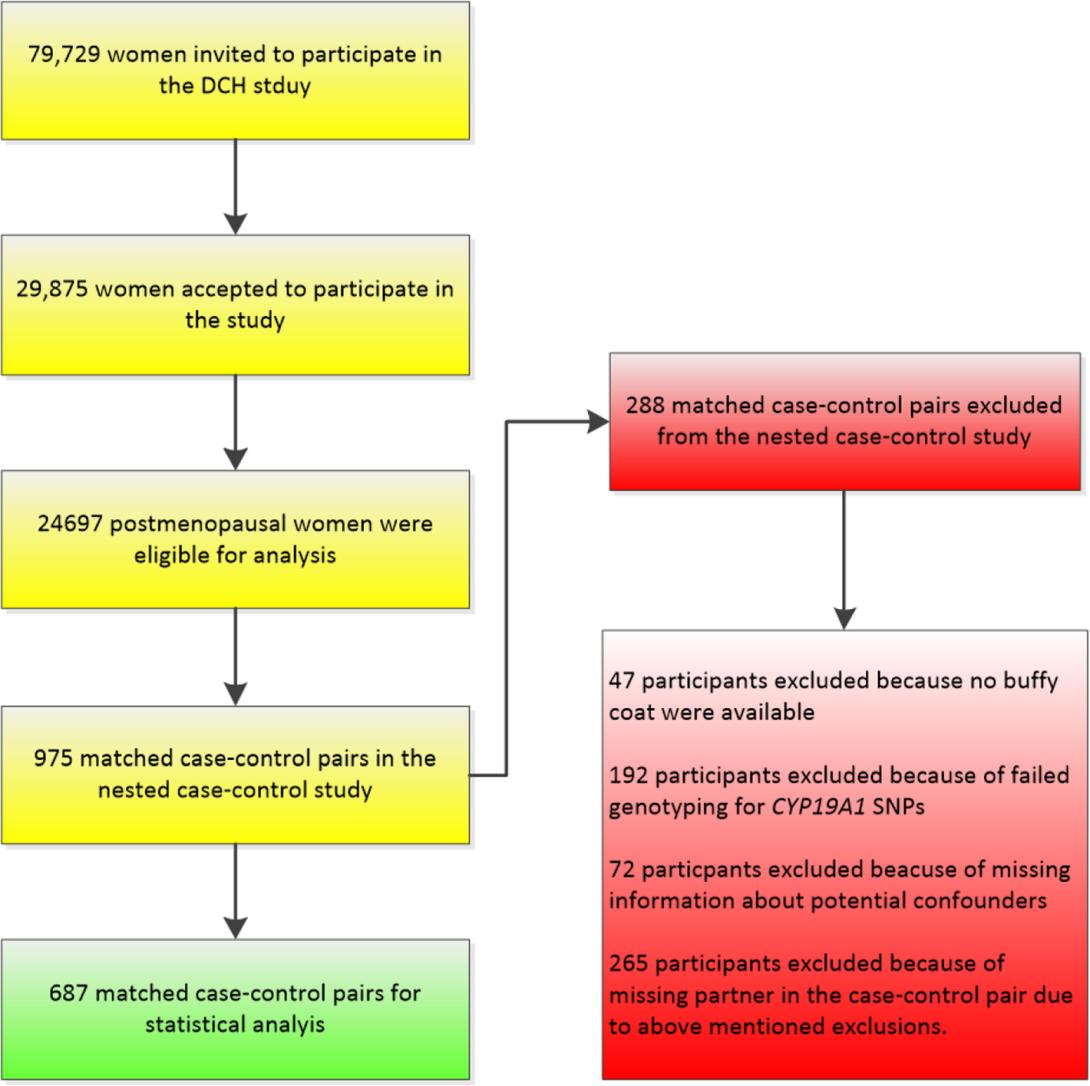
Flow chart illustrating inclusion and exclusions of participants in the nested case-control postmenopausal DCH study

Study participants were followed up for diagnosis of BC from date of entry until either the date of diagnosis of cancer using record linkage to the Danish Cancer Registry until 2003 and afterwards by linkage to the Danish Pathology Databank, date of death, date of emigration, or April 27th, 2006, whichever came first. A total of 975 women were diagnosed with BC during the follow-up period. For each case, one matched control was selected [8, 30, 43]. The control was cancer-free at the exact age at diagnosis of the case and was further matched on age at inclusion into the cohort (half-year intervals), use of hormone replacement therapy (HRT) (current/former/never) and on certainty of postmenopausal status (known/probably postmenopausal) upon inclusion into the cohort. “Known” postmenopausal status was defined as women that were either: (1) non-hysterectomized and reporting no menstruation during 12 months before inclusion, (2) reporting bilateral oophorectomy, or (3) reporting age at last menstruation lower than age at hysterectomy. “Probably” postmenopausal status was defined as women that were either: (1) reporting menstruation during the 12 months prior to inclusion and current use of HRT (we assumed the bleeding was caused by HRT), (2) reporting hysterectomy with a unilateral oophorectomy or an oophorectomy of unknown laterality, or (3) reporting last menstruation at the same age as age of the hysterectomy operation [8]. 72 individuals were excluded because of missing information about one or more of the potential confounding variables. Additionally 239 individuals were excluded because of failed genotyping or no buffy coat was available. 265 individuals were excluded because of a missing partner in the matched case-control pair, due to the above mentioned exclusions leaving 687 pairs for data analyses (Fig. 1).

Levels of estrone, estrone sulphate and SHBG were previously determined for a subset of the DCH study (434 cases and controls) in plasma samples collected at entry into the cohort - a cross-sectional study [44]. Of these 434 cases and controls, 8 had extreme hormone measures, 117 had missing values on hormone analyses, genotypes or confounder information, and 404 were present users of HRT resulting in 339 women for these analyses.

#### Data on covariates

From the baseline questionnaires we obtained information on duration of school education, smoking status, HRT use, birth pattern (number of births and age at first birth) and alcohol intake. Body Mass Index (BMI) was computed based on measurements of height and weight at enrolment (kg/m^2^). Intake of alcohol was inferred from the food-frequency questionnaire and life-style questionnaire as described in details in [8, 30]. Abstainers were defined as those who reported no intake of alcohol on the food-frequency questionnaire and no drinking occasions on the lifestyle questionnaire. The lifestyle questionnaire included this question regarding use of NSAID: Have you taken more than one pain relieving pill per month during the last year? If the answer was yes, the participant was asked to record how frequent they took each of the following medications: ‘aspirin’, ‘paracetamol’, ‘ibuprofen’, or ‘other pain relievers’. The latter category included NSAID preparations other than aspirin and ibuprofen. Based on all records, we classified study subjects according to use of ‘any NSAID’ (≥2 pills per month during one year) at baseline. Findings on all of these known risk factors have been reported previously for both the entire DCH cohort, for a subset of the present study, and for the present study group [5, 8, 29, 42, 45, 46].

#### Ethics statement

All participants gave verbal and written informed consent before enrolment to the study. The Diet, Cancer and Health study was approved by the National Committee on Health Research Ethics (journal nr. (KF) 01-345/93), and the Danish Data Protection Agency.

### RCT

#### Participants

The RCT was conducted at the Department of Nutrition, Exercise and Sports, University of Copenhagen, Denmark. The participants were recruited by advertisements in the Copenhagen area, in local newspapers and at the webpage www.forsogsperson.dk. To be eligible, women had to meet the following requirements: 1) aged 50–70 years and postmenopausal (last menstruation at least 1 year earlier); 2) having no history of hysterectomy *before* last menstruation *with* preservation of both ovaries (unless a medical confirmation for the postmenopausal status exists or the participant is 60 years or older); 3) having no major health problems, such as ulcers, heart diseases, diabetes or cancer; 4) having a weekly alcohol use of less than 14 drinks, but not being an abstainer and having no history of alcohol abuse; 5) not using HRT; 6) not taking prescription medications that could interfere with the study (i.e. daily use of NSAIDs and/or medication that interact with PPARγ e.g. cholesterol lowering medicine); 7) having a BMI of 18–35; 8) not being allergic to alcohol and/or Ibuprofen; 9) being a non-smoker.

Power calculation showed that there was an 80 % chance of finding a 10 % change in estradiol level if 11 participants were included in each group (α = 5 %). In order to take drop out into account, we decided to enrol 18 women in each group. However, only 7 (16 %) women were *PPARG*^12^Ala carriers, and they were all included in the study as well as 18 homozygous wild type carriers of the *PPARG* Pro^12^ allele.

#### Study design

**Fig. 2:**
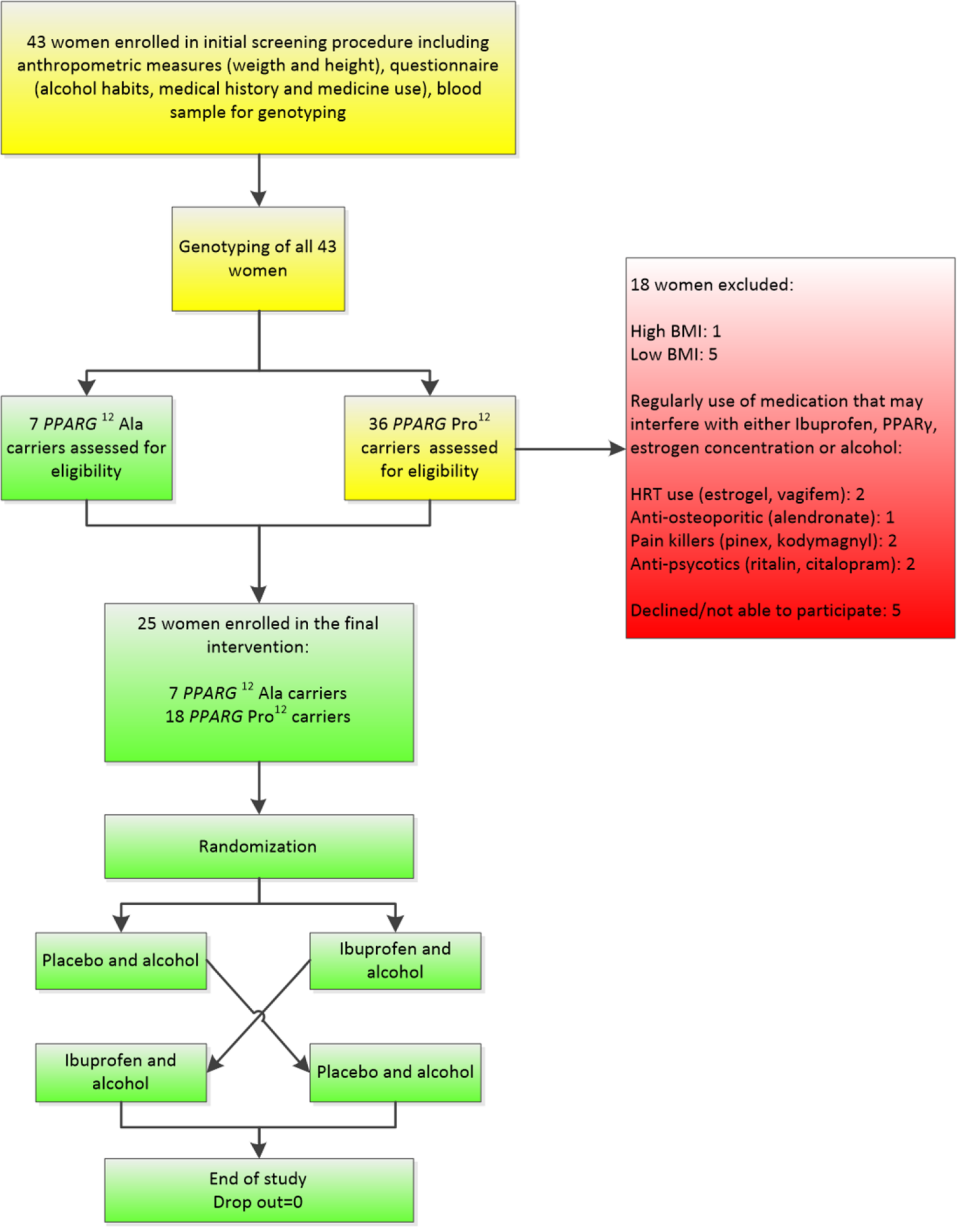
Flow chart illustrating inclusion and exclusions of participants in the RCT performed as a randomised, double-blinded, placebo controlled 2x24 h crossover study

The study was performed as a randomised, double-blinded, placebo controlled 2x24 h crossover study as illustrated in Fig. 2. The two interventions were separated by a 5–7 week washout period. Alcohol was supplied as 96 % ethanol (Navimer, G.D.C., Jumet, Belgium) in an 8 % solution with 1:8 Rose’s Lime Flavour Cordial Mixer® (Dr Pepper Snapple Group Inc., Plano, Texas), 1:8 Rose’s Sugar Cane Flavour Cordial Mixer® (Dr Pepper Snapple Group Inc., Plano, Texas) and water (0.4 g ethanol/kg b.w.). Placebo and Ibuprofen tablets looked identical. Placebo tablets were supplied by The Pharmacy of the Capital Region of Denmark (Herlev, Denmark), and Ibuprofen (2 x 200 mg) by Nycomed ApS (Roskilde, Denmark).

**Fig. 3:**
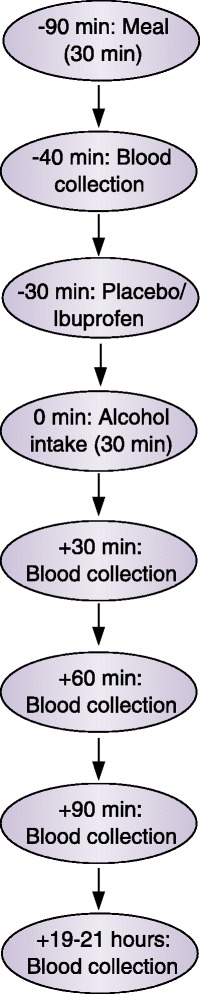
The study course for each participant at each intervention in the RCT. The intervention was repeated with or without Ibuprofen resulting in two interventions per participant

Forty-eight hours before each intervention, participants were asked to refrain from alcohol consumption and any form of painkillers. The participants showed up fasting and were served lunch at the University’s dining facility 1½ hour before drinking (Fig. 3). The lunch consisted of a sandwich, which was identical for each participant at each intervention, and was eaten within 30 min. All blood samples were collected following 10 min supine resting. The first blood sample was drawn 40 min before serving the drink. Just after this blood collection and 30 min prior to drinking, participants had their Ibuprofen or placebo tablet administered. This time point was chosen in order to capture the plasma concentration peak of Ibuprofen which is 1–2 h after administration [47, 48]. After another 30 min, the alcoholic beverage was administered and consumed within 30 min under the surveillance of a research assistant to ensure that the drink was ingested slowly over the entire period. After 30, 60 and 90 min, blood was collected. These time points were selected based on two other studies reporting peak in blood estradiol concentrations between 30 and 60 min after alcohol consumption [16, 20]. The next morning, the participants showed up again for the last blood collection. This time point was chosen to enable the comparison of our results with those obtained in long term intervention or cohort studies where blood typically is collected the day after having consumed alcohol. Since estrone sulphate has a half-life of 5–7 h [17], an alcohol induced change in estrone sulphate caused by disruption in estrogen biosynthesis would be measurable in this morning blood sample.

#### Blood sampling and storage

For the genotype screening, blood was collected in 6 ml EDTA BD Vacutainer® Blood Collection Tubes (Becton, Dickinson and Company) and buffy coat was separated by centrifugation (4000 rpm, 4 °C) and frozen at −80 °C. During the intervention, blood was collected in 10 ml silicone-coated serum BD Vacutainer® Blood Collection Tubes (Becton, Dickinson and Company) for ethanol and hormone measurements. The tubes were left at room temperature for >30 min, centrifuged (4000 rpm, 4 °C) and aliquots were frozen at −80 °C. To avoid ethanol evaporation from the tubes during storage, serum for ethanol analysis was kept in tubes with airtight caps.

#### Ethics statement

The research protocol was approved by The National Committee on Health Research Ethics for the Capital Region of Denmark, protocol number: H-3-2013-056. All participants received oral and written information concerning the study before giving their written consent.

### Laboratory methods

#### DCH cohort study

DNA from the DCH participants was extracted from frozen lymphocytes as described [49]. *PPARG* Pro^12^Ala (rs1801282), *PPARGC1A* Gly^482^Ser (rs8192678) and *PPARGC1A* Thr^612^Met (rs3736265) were determined as previously described [30] and have been published previously [29, 30]. Ten tagging SNPs of the *CYP19A1* gene were selected using publicly available HapMap [50] genotyping data set from Utah residents with ancestry from Northern and Western Europe (CEU, version 3, release 2) in combination with Haploview (version 4.2, Broad Institute, Cambridge USA) [51] comprising the major variations in *CYP19A1*. Criteria for SNP inclusions were a minor allele frequency of minimum 5 % and an r^2^ threshold of 0.1. Tagging of SNPs with an r^2^ higher than 0.1 resulted in several SNPs being in complete linkage disequilibrium. Moreover, our intention was to include only major polymorphisms in the gene. Four polymorphisms were force included in the aggressive SNP tagging (rs10046, rs749292, rs1062033 and rs10519297) due to their documented effect on aromatase RNA expression, blood hormone levels and/or BC risk [11, 52–59]. Moreover, one of the tag SNPs (rs4646) has been associated with BC [59] and blood estrogen levels [53, 56, 60]; another tag SNP (rs2445762) was associated with low estradiol levels in GWAS [61]; and rs3751591 is located in a recombination spot between two haplotype blocks (Additional file 1).

Four of the polymorphisms (rs10046, rs749292, rs1062033 and rs10519297) were determined using the ABI 7900HT RT-PCR system (Applied Biosystems, Nærum, Denmark): rs10046 and rs1062033 were determined using the TaqMan® Pre-designed SNP genotyping; assay ID C_8234732_30 and C_8794680_30, respectively (Applied Biosystems, Nærum, Denmark). rs749292: Primers: F: 5’-GCT TCT GCC AGT CCT TCT TCA-3’, R: 5’-GCT TAG GGC CTG ATA GAA ATT GTG-3’ (TAGCopenhagen, Copenhagen, Denmark), G-allele: 5’-FAM-CTC GGA GTC GAG GAT-MGB-3’, A-allele: 5’-VIC-TCG GAG TCA AGG ATT-MGB-3’ (Applied Biosystems, Nærum, Denmark). rs10519297: Primers: F: 5’CCT TGC CTG AGC CAT CTC TT-3’, R: 5’- CTT GGC AGT CAA AAG CAG TAG TAG TC-3’ (TAGCopenhagen, Copenhagen, Denmark), G-allele: 5’-FAM-CTC CGA CAT GGG TC-MGB-3’, A-allele: 5’-VIC-TCT CCG ACA TAG GTC-MGB-3’ (Applied Biosystems, Nærum, Denmark).

The remaining six *CYP19A1* polymorphisms (rs2008691, rs2445762, rs3751591, rs4646, rs6493487 and rs749292) were genotyped by KBioscience (KBioscience, Hoddesdon, United Kingdom) by PCR-based KASP™ genotyping assay (http://www.lgcgenomics.com/). To confirm reproducibility, genotyping was repeated for 10 % of the samples yielding 100 % identical genotypes.

Serum levels of estrone, estrone sulphate and sex-hormone binding globulin (SHBG) were previously determined for a subset of the DCH study (434 cases and controls) in blood samples collected at entry into the DCH cohort [44]. Estrone and estrone sulphate were measured by radioimmunoassay and SHBG by use of immunofluorometric analysis.

#### RCT

DNA was isolated from frozen lymphocytes as described by Miller et al. [49]. Genotypes were determined using RT-PCR and allelic discrimination on ABI 7900HT instruments (Applied Biosystems, Nærum, Denmark). Generally, 40–200 ng/μl DNA was obtained and 10 ng of DNA was genotyped in five μl containing 50 % 2 × Mastermix (Applied Biosystems, Nærum, Denmark), 100 nM probes, and 900 nM primers. *PPARG* Pro^12^Ala (rs1801282) primers and probes were: primers: 5’-GTT ATG GGT GAA ACT CTG GGA GAT-3’ and 5’-GTT ATG GGT GAA ACT CTG GGA GAT-3’: probes: C-allele: 5’‘-FAM-CTC CTA TTG ACG CAG AAA GCG ATT C-BHQ-1-3’ and G-allele: 5’-Yakima Yellow-TCC TAT TGA CCC AGA AAG CGA TTC C-BHQ-1-3’. Serum ethanol concentration was measured by the enzymatic rate assay (kat. no. 11776312190, Roche Diagnostics GmbH, Mannheim, Germany) using the ABX Pentra 400 (Horiba Medical, Brøndby, Denmark) instrument with inter- and intra assay variations of <4 %. All samples from each participant were analysed in duplicates in random order and in the same batch to decrease analytical variation.

Analyses of estrogens and SHBG levels were performed at the Department of Clinical Biochemistry and Immunology, Statens Serum Institute, Copenhagen, Denmark. Estrogens were extracted by Solid-Phase Extraction and the extracts were analyzed using Liquid-Chromatography (LC) coupled to Mass-Spectrometry (MS). All samples were analyzed in duplicate and the average of both measurements was used. Within the working range of the method (20 pmol/L to 50 nmol/L) the coefficient of variation (CV) for all analytes were 10 % for estrone, estrone sulphate and 17β-estradiol and the CV’s increased below the working range and reached 20 % at the limit of quantification (LOQ) (10 pmol/L). 60 % of the estradiol analyses were below the limit of detection (LOD) (1 pmol/L); therefore, these measurements were excluded from statistical analyses. SHBG was determined on an Abbott Architect using Abbotts SHBG kit (Abbott, Abbott Park, Illinois). The LOD and the intra- and inter-assay CV were 0.1 nmol/l, 4 and 6 %, respectively.

### Statistical methods

#### DCH cohort study

Deviation from Hardy-Weinberg equilibrium was assessed using a Chi square test.

The analysis of the association between the exposure variables and the BC incidence rate ratios (IRR) was based on a conditional logistic regression analysis corresponding to a Cox Proportional Hazard model due to the used study design [62]. Age was used as the time axis. Two-sided 95 % confidence intervals (CI) for the IRR were calculated on the basis of Wald’s test of the regression parameter, that is, on the log rate ratio scale. All models were adjusted for baseline values of risk factors for BC such as parity (entered as two variables; parous/nulliparous and number of births), age at first birth, length of school education (low, medium and high), duration of HRT use, and BMI. Analyses not concerning interaction between alcohol intake and the polymorphisms were further adjusted for alcohol intake of 10 g per day. For the different genetic variations, we investigated different interactions with alcohol intake and use of NSAID, using the likelihood ratio test. IRR was calculated separately for heterozygous and homozygous variant allele carriers. For all the SNPs except for rs3751591, all variant allele carriers were subsequently grouped for interaction analyses since no recessive effects were observed. For rs3751591, a recessive mode was used in the subsequent analyses. Haplotypes of *CYP19A1* were inferred manually as done previously [63–65].

For the analyses with sex-hormone levels as outcome, multiple log-linear regression analyses of the association between *CYP19A1* genotypes and serum levels of estrone, estrone sulphate and SHBG were performed with adjustment for potential confounders i.e., age (linear), smoking (categorical: never, past and current) and BMI (linear). All interaction terms were kept in the models in all analyses even though there was no interaction for comparison. All values of hormone concentrations were log-transformed to correct for left-skewed distribution. The statistical analyses were carried out using the PHREG and GLM procedure in SAS (release 9.3, SAS Institute, Inc, Cary, North Carolina, USA).

#### RCT

Linear regression was used to evaluate the effect on *PPARG* Pro^12^Ala genotype status and treatment of Ibuprofen during consumption of alcohol.

Two different models were used to analyse the data due to non-linear responses with time over the whole period (0 to 1200 min). Model A only involved the time at the beginning of the trial period and the end (time = 0 and time = 1200 min), whereas model B involved the times 0, 30, 60, and 90 min after start. Model A used the PROC MIXED procedure in SAS with *id* as the random effect nested with genotype and order (geno*order). The explanatory categorical variables were treatment with Ibuprofen and placebo (*treat*), order in which they were given Ibuprofen or placebo (*order*), genotype (*geno*), and *time* (0 and 1200). *BMI* was included as a covariate.

Model B also used the PROC MIXED procedure in SAS with *id* as the random effect nested with genotype and order (geno*order). The explanatory categorical variables were treatment with Ibuprofen or placebo (*treat*), order in which they were given Ibuprofen and placebo (*order*), genotype (*geno*), and *time* (0, 30, 60, 90). *BMI* was included as a covariate. *Time* was also included as repeated measurement with *id* (geno*treat*order) as the subject.

The response variable, estrone sulphate, was log-transformed to correct for right-skewed distribution. The response variables estrone, SHBG and ethanol were not log-transformed, since the conditional residuals were not or only very little skewed.

In some of the analyses, one or two outliers were removed if they were very dominating, had high Cook’s D value and a relatively large residual value. Zero values of estrone measurements (11 %) were replaced by half of LOD = 0.5 pmol/L.

The statistical analyses were carried out using SAS (release 9.3, SAS Institute, Inc, Cary, North Carolina, USA). For all tests, a *P* value less than 0.05 was considered statistically significant.

## Results

### DCH cohort study

Baseline characteristics of the present study group including BC risk factors are presented in Table 1 as published previously [8, 30]. Among the controls, the genotype distributions of the studied polymorphisms were in Hardy–Weinberg equilibrium (results not shown).

**Table 1:** Baseline characteristics of the DCH study participants by selected demographic and established BC risk factors

Variable	Cases		Controls		IRR^a^ (95 % CI)
	*n* (%)	Median (5–95 %)	*n* (%)	Median (5–95 %)	
Women	687 (100)		687 (100)		
Age at inclusion, years		57 (51–64)		57 (51–64)	
School education					
Short	198 (29)		257 (37)		1.0 (ref.)
Medium	344 (50)		316 (46)		1.39 (1.07–1.79)
Long	145 (21)		114 (17)		1.59 (1.13–2.24)
Body mass index, kg/m^2^		25 (20–34)		25 (20–34)	1.01 (0.96–1.07)^b^
Nulliparous	102 (15)		78 (11)		1.02 (0.64–1.60)^c^
Number of births		2 (1–4)		2 (1–4)	0.92 (0.79–1.06)
Age at first birth, years		23 (18–31)		23 (18–32)	1.07 (0.91–1.25)^d^
Use of HRT, years^e^		6 (0.5–19)		5 (0.5–20)	1.00 (0.87–1.15)^f^
Abstainers	15 (2)		22 (3)		0.80 (0.40–1.61)^g^
Alcohol intake, g/day		11 (1–43)		9 (1–40)	1.12 (1.04–1.21)^h^
NSAID use^i^	286 (42)		239 (35)		1.33 (1.07–1.66)

Values are expressed as medians (5th and 95th percentiles) or as fractions (%)

^a^The risk estimates for breast cancer are mutually adjusted

^b^The risk is estimated per additional 2 kg/m^2^

^c^The risk is estimated for nulliparous versus one birth at age 35

^d^The risk is estimated per additional 5 years

^e^Among ever users of HRT

^f^The risk is estimated per additional 5-year of HRT use

^g^The risk for abstainers compared to the increment of 10 g alcohol per day

^h^Among drinkers, risk estimate is estimated for the increment of 10 g alcohol per day

i NSAID use is defined as ≥ 2 pills per month during one year

#### Associations with hormone levels

The cross-sectional study group including 339 women from the matched case-control study was used for this analysis. Among past and never users of HRT, the hormone levels were associated with genotype of five of the ten studied polymorphisms. Variant T-carriers of the *CYP19A1*/rs11070844 polymorphism had 17 % higher estrone levels (*P* = 0.009) and 14 % higher estrone sulphate levels (*P* = 0.01) than homozygous wild type allele carriers (Table 2). SHBG levels were 37 % higher among CC-carriers of the *CYP19A1*/rs3751591 polymorphisms compared to T-carriers (*P* = 0.03). Carriers of the variant alleles of the two *CYP19A1* polymorphisms rs749292 and rs1062033 had 12 % lower levels of estrone sulphate compared to homozygous wild type allele carriers (*P* = 0.004 and 0.007, respectively), and variant carriers of the *CYP19A1*/rs10519297 polymorphism had 12 % higher levels of estrone sulphate compared to the wild type (*P* = 0.03). Thus, several of the studied SNPs were associated with hormone levels (Table 2).

**Table 2:** Plasma levels of estrone, estrone sulphate and SHBG among 339 never and past users of HRT as percentage difference in hormonal measurements in relation to *CYP19A1* polymorphisms

Genotype	*n* (%)	Estrone	*P*-value^b^	Estrone sulphate	*P*-value^b^	SHBG	*P*-value^b^
	*n* = 339	∆ (95 % CI)^a^		∆ (95 % CI)^a^		∆ (95 % CI)^a^	
rs10519297							
AA	81 (24)	0 (ref.)		0 (ref.)		0 (ref.)	
AG	187 (55)	8 (−4;21)	0.35	10 (−1;21)	0.05	−5 (−14;6)	0.66
GG	71 (21)	1 (−13;16)		16 (3;31)		−2 (−14;11)	
AG + GG	258 (56)	6 (−6;18)	0.34	12 (1;23)	0.03	−4 (−13;6)	0.44
rs749292							
GG	101 (30)	0 (ref.)		0 (ref.)		0 (ref.)	
AG	169 (50)	−5 (−15;6)	0.62	−11 (−19;-2)	0.007	−3 (−12;7)	0.07
AA	69 (20)	−5 (−17;9)		−16 (−26;-6)		11 (−2;26)	
AG + AA	238 (70)	−5 (−14;5)	0.33	−12 (−20;-4)	0.004	1 (−8;11)	0.82
rs1062033							
CC	88 (26)	0 (ref.)		0 (ref.)		0 (ref.)	
CG	180 (53)	−2 (−13;10)	0.53	−10 (−19;-1)	0.01	−1 (−11;9)	0.45
GG	71 (21)	−7 (−20;6)		−16 (−26;-6)		6 (−7;21)	
CG + GG	251 (74)	−4 (−14;7)	0.51	−12 (−20;-3)	0.007	1 (−9;11)	0.88
rs10046							
AA	93 (27)	0 (ref.)		0 (ref.)		0 (ref.)	
AG	177 (52)	2 (−9;15)	0.58	4 (−5;15)	0.26	−1 (−10;10)	0.94
GG	69 (20)	−4 (−17;10)		11 (−2;25)		−2 (−14;11)	
AG + GG	246 (73)	0 (−10;12)	0.93	6 (−3;16)	0.21	−1 (−10;9)	0.82
rs4646							
CC	186 (55)	0 (ref.)		0 (ref.)		0 (ref.)	
CA	133 (39)	3 (−7;14)	0.68	5 (−3;15)	0.38	−4 (−13;5)	0.59
AA	20 (6)	−6 (−23;16)		10 (−8;31)		−5 (−21;15)	
CA + AA	153 (45)	2 (−8;12)	0.73	6 (−3;15)	0.18	−4 (−13;4)	0.30
rs6493487							
AA	203 (60)	0 (ref.)		0 (ref.)		0 (ref.)	
GA	127 (38)	−5 (−14;5)	0.27	4 (−4;14)	0.63	−5 (−13;4)	0.37
GG	9 (3)	−19 (−40;9)		0 (−23;29)		−14 (−34;13)	
GA + GG	136 (41)	−6 (−15;4)	0.23	4 (−4;13)	0.36	−5 (−13;3)	0.22
rs2008691							
AA	220 (65)	0 (ref.)		0 (ref.)		0 (ref.)	
GA	109 (32)	4 (−6;15)	0.35	3 (−6;12)	0.83	2 (−7;12)	0.17
GG	10 (3)	21 (−9;60)		0 (−21;28)		27 (−1;64)	
GA + GG	119 (35)	5 (−4;16)	0.29	2 (−6;12)	0.57	4 (−5;14)	0.37
rs3751591							
TT	241 (71)	0 (ref.)		0 (ref.)		0 (ref.)	
TC	90 (27)	2 (−9;13)	0.55	1 (−8;10)	0.50	1 (−9;11)	0.09
CC	8 (2)	19 (−13;63)		18 (−10;54)		38 (4;83)	
CC vs. TT + TC	8 (2)	18 (−14;62)	0.29	17 (−10;53)	0.24	37 (4;82)	0.03
rs2445762							
TT	175 (52)	0 (ref.)		0 (ref.)		0 (ref.)	
TC	142 (42)	−3 (−12;7)	0.71	−1 (−9;8)	0.71	−3 (−19;16)	0.53
CC	22 (6)	−7 (−24;13)		−7 (−21;10)		−5 (−13;4)	
TC + CC	164 (48)	−3 (−12;6)	0.47	−2 (−10;6)	0.63	−5 (−13;4)	0.27
rs11070844							
CC	267 (79)	0 (ref.)		0 (ref.)		0 (ref.)	
TC	68 (20)	17 (4;31)	0.03	13 (2;25)	0.04	6 (−5;18)	0.20
TT	4 (1)	16 (−26;79)		23 (−16;79)		36 (−9;103)	
TC + TT	72 (21)	17 (4;31)	0.009	14 (3;25)	0.01	7 (−3;19)	0.18

*SHBG* Sex-hormone binding globulin

Δ Percentage difference in hormonal measurements compared to WT

^a^Adjusted for age, smoking (never, past, current), alcohol intake (increment of 10 g per day) and BMI (kg/m^2^) at baseline

^b^ *P*-value for trend

Three of the ten *CYP19A1* polymorphisms were associated with alcohol-dependent changes in hormone levels according to genotype (Table 3). Carriers of the variant alleles of *CYP19A1*/rs2008691 and *CYP19A1*/rs1062033 polymorphisms had 3 % and 1 % higher estrone sulphate levels, respectively, compared to homozygous wild type carriers (*P*-*value for interaction* (*P*_*int*_) = 0.02 and 0.03, respectively) per 10 g alcohol intake per day. Variant T-carriers of the *CYP19A1*/rs11070844 polymorphism had 3 % lower levels of SHBG compared to the wild type carriers (*P*_*int*_ = 0.03) per 10 g daily alcohol intake. In general, estrone and estrone sulphate levels increased whereas SHBG levels decreased for every 10 g alcohol consumed per day irrespectively of genotype (Table 3). Estrone sulphate levels differed significantly according to *CYP19A1*/rs3751591 genotype for NSAID users and non-users, respectively (*P*_*int*_ = 0.008) (Additional file 2). Carriers of the CC genotype had 48 % higher levels of estrone sulphate when using NSAID compared to T-carriers who did not use NSAID (95 % CI: 3;114), whereas T-allele carriers who were also NSAID users had 13 % decreased levels of estrone sulphate (95 % CI; −20;-5). However, these estimates were based on very small numbers. There was also a borderline statistically significant interaction between NSAID usage and the *CYP19A1*/rs6493487 polymorphism in relation to SHBG levels (*P*_*int*_ = 0.05). Overall, NSAID users had higher levels of SHBG and lower levels of estrone and estrone sulphate (Additional file 2). Thus, some of the studied *CYP19A1* SNPs were associated with hormone levels and a few other SNPs interacted with alcohol intake in relation to hormone levels. *CYP19A1*/rs1062033 was associated to both hormone levels and to alcohol-dependent differences in hormone levels.

**Table 3:** Plasma levels of estrone, estrone sulphate and SHBG among 325 never and past users of HRT, who were also current drinkers, as percentage difference in hormonal measurements per 10 g/day in alcohol intake

Genotype	*n* (%)	Estrone	*P*-value^b^	Estrone sulphate	*P*-value^b^	SHBG	*P*-value^b^
	*n* = *325*	∆ (95 % CI)^a^		∆ (95 % CI)^a^		∆ (95 % CI)^a^	
rs10519297							
AA	74 (23)	2 (−4;9)		3 (−3;9)		−5 (−11;0)	
AG	182 (56)	3 (−1;6)	0.82	3 (0;7)	0.17	−6 (−9;-3)	0.23
GG	69 (21)	1 (−5;6)		4 (0;9)		−3 (−7;2)	
AG + GG	251 (77)	2 (−1;5)	0.54	4 (1;6)	0.08	−5 (−8;-2)	0.27
rs749292							
GG	98 (30)	3 (−2;7)		4 (1;8)		−5 (−8;-1)	
AG	165 (51)	1 (−3;6)	0.96	3 (0;7)	0.38	−6 (−9;-2)	0.94
AA	62 (19)	2 (−5;10)		−1 (−7;6)		0 (−7;6)	
AG + AA	227 (70)	1 (−2;5)	0.85	3 (−1;6)	0.15	−5 (−8;-1)	0.95
rs1062033							
CC	86 (26)	1 (−3;6)		4 (0;8)		−5 (−9;-1)	
CG	175 (54)	3 (−1;7)	0.76	4 (0;7)	0.11	−5 (−8;-2)	0.67
GG	64 (20)	1 (−6;8)		1 (−4;7)		−4 (−9;2)	
CG + GG	239 (74)	2 (−1;6)	0.45	3 (0;7)	0.03	−5 (−8;-2)	0.90
rs10046							
AA	85 (26)	3 (−3;10)		4 (−2;10)		−7 (−12;-1)	
AG	173 (53)	2 (−1;7)	0.89	3 (0;7)	0.29	−6 (−9;-3)	0.06
GG	67 (21)	1 (−4;5)		3 (0;8)		−3 (−7;1)	
AG + GG	240 (74)	2 (−1;5)	0.64	3 (1;6)	0.22	−5 (−7;-2)	0.24
rs4646							
CC	177 (54)	3 (−2;7)		4 (0;7)		−4 (−8;-1)	
CA	129 (40)	2 (−2;6)	0.49	3 (0;7)	0.30	−5 (−8;-2)	0.87
AA	19 (6)	−6 (−18;7)		6 (−5;19)		−8 (−18;4)	
CA + AA	148 (46)	1 (−2;5)	0.41	3 (0;7)	0.14	−5 (−8;-2)	0.59
rs6493487							
AA	192 (59)	3 (−1;8)		3 (−1;7)		−5 (−9;-1)	
GA	125 (39)	2 (−2;5)	0.79	4 (1;8)	0.64	−5 (−8;-2)	0.60
GG	8 (2)	−1 (−11;11)		−1 (−10;9)		−5 (−14;5)	
GA + GG	133 (41)	1 (−2;5)	0.79	4 (1;7)	0.97	−5 (−8;-2)	0.33
rs2008691							
AA	210	2 (−2;6)		5 (2;8)		−6 (−9;-3)	
	(65)						
GA	106 (32)	2 (−2;6)	0.67	1 (−2;5)	0.03	−4 (−8;0)	0.41
GG	9 (3)	12 (−4;31)		10 (−3;26)		6 (−7;22)	
GA + GG	115 (35)	2 (−2;7)	0.70	2 (−2;6)	0.02	−4 (−7;0)	0.31
rs3751591							
TT	232 (72)	2 (−1;6)		4 (1;7)		−5 (−8;-2)	
TC	85 (26)	1 (−3;6)	0.78	2 (−2;6)	0.70	−5 (−8;-1)	0.90
CC	8 (2)	11 (−7;31)		9 (−6;26)		10 (−6;28)	
CC vs. TT + TC	8 (2)	11 (−7;31)	0.81	9 (−6;26)	0.74	10 (−6;28)	0.86
rs2445762							
TT	166 (51)	2 (−1;6)		3 (0;6)		−4 (−7;-1)	
TC	139 (43)	1 (−3;6)	0.94	5 (1;9)	0.19	−6 (−10;-2)	0.72
CC	20 (6)	3 (−9;16)		−2 (−11;8)		−8 (−17;2)	
TC + CC	159 (49)	1 (−3;6)	1.00	4 (0;8)	0.17	−6 (−10;-3)	0.61
rs11070844							
CC	255 (78)	1 (−2;5)		3 (0;6)		−4 (−7;-1)	
TC	66 (21)	5 (−1;11)	0.73	7 (1;12)	0.92	−7 (−12;-3)	0.06
TT	4 (1)	4 (−20;37)		15 (−9;45)		11 (−13;42)	
TC + TT	70 (22)	5 (−1;11)	0.42	7 (2;12)	0.74	−7 (−12;-2)	0.03

*SHBG* Sex-hormone binding globulin

ΔPercentage difference in hormonal measurements per 10 g/day difference in alcohol intake

^a^Adjusted for age, smoking (never, past, current) and BMI (kg/m^2^) at baseline

^b^P-value for interaction. All interactions were kept in the models in all analyses even though there was no interaction

#### Associations with BC risk

Homozygous variant carriers of the *CYP19A1*/rs3751591 polymorphism were at 2.12-fold increased risk of BC (95 % CI: 1.02-4.43) compared to wild-type carriers (Table 4). Carriers of the haplotype combination GGG/GAG (*CYP19A1*/A-rs10046-G, A-rs6493487-G, A-rs10519297-G) were at 56 % increased risk of BC (IRR = 1.56; 95 % CI: 1.02-2.40) (Additional file 3). Thus, *CYP19A1*/rs3751591 was both associated with SHBG levels and with risk of BC such that homozygous variant allele carriers had higher levels of serum SHBG and were at increased risk of BC.

**Table 4:** IRR for BC in relation to the studied polymorphisms among postmenopausal women in the DCH cohort

	*n*_cases_ (%)	*n*_control_ (%)	IRR^a^ (95 % CI)	IRR^b^ (95 % CI)	*P*-value^c^
	(*n* = 687)	(*n* = 687)			
rs10519297					
AA	170 (25)	174 (25)	1.00 (ref.)	1.00 (ref.)	
AG	341 (50)	361 (53)	0.98 (0.76–1.27)	0.94 (0.72–1.23)	0.15
GG	176 (25)	152 (22)	1.23 (0.90–1.68)	1.25 (0.91–1.72)	
AG + GG	511 (75)	513 (54)	1.05 (0.82–1.34)	1.03 (0.80–1.32)	0.83
rs749292					
GG	216 (31)	203 (30)	1.00 (ref.)	1.00 (ref.)	
AG	332 (48)	352 (51)	0.89 (0.69–1.14)	0.89 (0.69–1.16)	0.62
AA	139 (20)	132 (19)	0.97 (0.71–1.33)	0.99 (0.72–1.37)	
AG + AA	471 (68)	484 (70)	0.91 (0.72–1.15)	0.92 (0.72–1.17)	0.50
rs1062033					
CC	203 (30)	186 (27)	1.00 (ref.)	1.00 (ref.)	
CG	333 (48)	354 (52)	0.85 (0.66–1.10)	0.85 (0.65–1.11)	0.46
GG	151 (22)	147 (21)	0.92 (0.67–1.25)	0.94 (0.68–1.28)	
CG + GG	484 (70)	501 (73)	0.87 (0.68–1.11)	0.88 (0.68–1.12)	0.30
rs10046					
AA	182 (27)	188 (28)	1.00 (ref.)	1.00 (ref.)	
AG	346 (50)	353 (51)	1.02 (0.79–1.31)	0.97 (0.75–1.26)	0.52
GG	159 (23)	146 (21)	1.16 (0.85–1.57)	1.15 (0.84–1.57)	
AG + GG	505 (73)	499 (72)	1.06 (0.84–1.34)	1.02 (0.80–1.31)	0.86
rs4646					
CC	372 (54)	371 (54)	1.00 (ref.)	1.00 (ref.)	
CA	265 (39)	262 (38)	1.00 (0.80–1.25)	0.97 (0.77–1.21)	0.95
AA	50 (7)	54 (8)	0.96 (0.63–1.45)	1.00 (0.65–1.53)	
CA + AA	315 (46)	316 (46)	0.99 (0.81–1.22)	0.97 (0.78–1.20)	0.78
rs6493487					
AA	407 (59)	430 (62)	1.00 (ref.)	1.00 (ref.)	
GA	245 (36)	218 (32)	1.23 (0.98–1.55)	1.24 (0.98–1.58)	0.16
GG	35 (5)	39 (6)	0.96 (0.60–1.54)	0.88 (0.54–1.44)	
GA + GG	280 (40)	257 (38)	1.19 (0.95–1.48)	1.18 (0.94–1.48)	0.16
rs2008691					
AA	479 (70)	470 (68)	1.00 (ref.)	1.00 (ref.)	
GA	179 (26)	198 (29)	0.88 (0.69–1.11)	0.88 (0.69–1.12)	0.25
GG	29 (4)	19 (3)	1.43 (0.80–2.57)	1.45 (0.80–2.64)	
GA + GG	208 (30)	217 (32)	0.93 (0.74–1.17)	0.93 (0.74–1.18)	0.57
rs3751591					
TT	479 (70)	498 (72)	1.00 (ref.)	1.00 (ref.)	
TC	182 (26)	176 (26)	1.07 (0.84–1.36)	1.07 (0.83–1.37)	0.60
CC	26 (4)	13 (2)	2.13 (1.04–4.39)	2.12 (1.02–4.43)	0.04
TC + CC	208 (30)	189 (28)	1.13 (0.89–1.42)	1.13 (0.89–1.44)	0.31
CC vs. TT + TC^d^	26 (4)	13 (2)	2.09 (1.02–4.29)	2.09 (1.00–4.34)	0.05
rs2445762					
TT	359 (52)	365 (53)	1.00 (ref.)	1.00 (ref.)	
TC	278 (40)	276 (40)	1.02 (0.81–1.27)	1.04 (0.83–1.31)	0.74
CC	50 (8)	46 (7)	1.10 (0.72–1.69)	1.19 (0.76–1.85)	
TC + CC	328 (48)	322 (47)	1.03 (0.83–1.27)	1.06 (0.85–1.32)	0.61
rs11070844					
CC	552 (80)	556 (81)	1.00 (ref.)	1.00 (ref.)	
TC	129 (19)	125 (18)	1.06 (0.801–1.39)	1.06 (0.80–1.40)	0.88
TT	6 (1)	6 (1)	1.01 (0.33–3.13)	0.86 (0.27–2.73)	
TC + TT	135 (20)	131 (19)	1.06 (0.81–1.38)	1.05 (0.80–1.38)	0.73

^a^Crude

^b^Adjusted for parous/nulliparous, number of births, age at first birth, length of school education (low, medium, high), duration of HRT use (years), BMI (kg/m^2^) and alcohol intake (10 g/day)

^c^ *P*-value for trend

^d^CC versus TT and TC

None of the *CYP19A1* polymorphisms interacted with alcohol (Additional file 4) or NSAID usage (Additional file 5) in relation to BC risk. All risk estimates showed increased risk of BC of 10-66 % per 10 g alcohol per day regardless of genotype (Additional file 4). NSAID usage also increased BC risk compared to non-users irrespectively of genotype (Additional file 5). There was no interaction between any of the *CYP19A1* polymorphisms and being carrier of either of the *PPARG* Pro^12^Ala alleles (Additional file 6). However, we found interaction between *CYP19A1*/rs3751591 and *PPARGC1A* Gly^482^Ser (*P*_*int*_ = 0.02) in relation to BC risk (Additional file 7); and interaction between *CYP19A1*/rs4646 and *PPARGC1A* Thr^612^Met (*P*_*int*_ = 0.002) in relation to BC risk (Additional file 8). Wild type carriers of *CYP19A1*/rs4646, who were also variant Met-carriers of *PPARGC1A* Thr^612^Met were at 2.06-fold increased risk of BC (95 % CI: 1.17-3.65). Conversely, variant *CYP19A1*/rs4646-carriers, who also carry the variant *PPARGC1A* Thr^612^Met allele had a 38 % decreased risk of BC (IRR = 0.62; 95 % CI: 0.36-1.08) (Additional file 8). When including alcohol in the model as 10 g alcohol per day, practically all *CYP19A1* polymorphisms interacted with *PPARG* Pro^12^Ala (*P*_*int*_-values between 0.03-0.10) (Additional file 9). Only *PPARG* Pro^12^Ala wild type carriers were at significantly increased risk of BC. Neither *PPARGC1A* Gly^482^Ser nor *PPARGC1A* Thr^612^Met interacted with any of the *CYP19A1* polymorphisms when inferring the BC risk per 10 g alcohol per day (Additional files 10 and 11). There were no interactions with NSAID use for combinations of *CYP19A1* genotypes for 10 g alcohol per day (Additional file 12).

### RCT

**Fig. 4:**
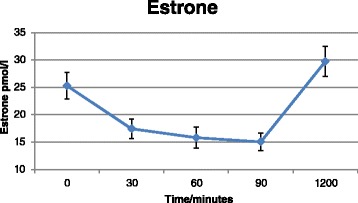
Estrone concentrations as a function of time. Zero values have been replaced by half of the Limit of Detection = 0.5 pmol/L. Values represent pooled mean measurements ± SEM (*n* = 50). *P*_time_ (0-90min) = <0.0001; *P*_time_ (0-1200min) = 0.02

**Fig. 5:**
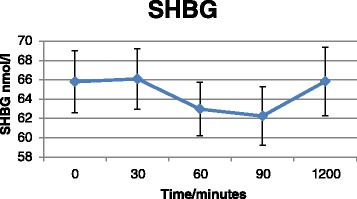
SHBG concentrations as a function of time. Values represent pooled mean measurements ± SEM (*n* = 50). *P*_time_ (0-90min) = 0.009

**Fig. 6:**
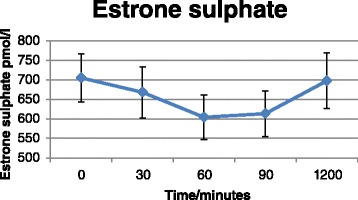
Estrone sulphate concentrations as a function of time. Values represent pooled mean measurements ± SEM (*n* = 50). *P*_time_ (0-90min) = <0.0001

**Fig. 7:**
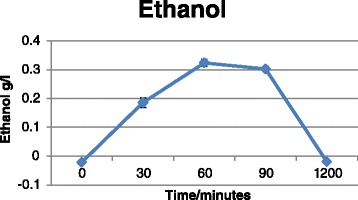
Ethanol concentrations as a function of time. Values represent pooled mean measurements ± SEM (*n* = 50). *P*_time_ (0-90min) = <0.0001

Baseline characteristics of the study participants are presented in Table 5, and mean hormone, ethanol and SHBG concentrations in Additional file 13. Baseline measurements (time = 0) did not differ between the two intervention groups (results not shown). Intake of Ibuprofen and *PPARG* Pro^12^Ala genotype were not associated with hormone or SHBG concentrations. However, there was a statistically significant effect of *time* on hormone concentrations (model B); that is, estrone, estrone sulphate and SHBG concentrations declined over the time period from 0 to 90 min (*P*_estrone_ = <0.0001, *P*_SHBG_ = 0.009 and *P*_estrone sulphate_ = <0.0001) (Figs. 4, 5 and 6), whereas the ethanol concentration increased as expected (*P*_ethanol_ = <0.0001) (Fig. 7). There was no effect of time in model A on any markers except for estrone concentrations, which increased at the latest time point (1200 min) compared to baseline (t = 0) (*P* = 0.02). BMI was significantly associated with concentrations of SHBG (*P* = 0.02) and ethanol (*P* = 0.04), such that women with high BMI also had higher blood concentrations.

**Table 5:** Baseline characteristics of the RTC study participants

Characteristic	*PPARγ2* Pro^12^ (*n* = 18)		*PPARγ2* ^12^Ala (*n* = 7)		*P*-values^d^
	Median	Range	Median	Range	
Age^a^, years	58.5	(49–70)	55	(47–67)	0.28
Weight, kg	64.1	(50.1–86.5)	59.8	(57.2–68.4)	0.27
BMI, kg/m^2^	23.5	(19.1–26.9)	22.4	(19.4–24.5)	0.14
Years since last menses	5.5	(1–25)	6	(3–16)	0.46
Alcohol intake/week	6.5	(1–15)	6	(3.5–14)	0.70
Characteristic	*PPARγ2* Pro^12^ (*n* = 18)		*PPARγ2* ^12^Ala (*n* = 7)		
	No.	%	No.	%	
Menopause type					
Natural^b^	17	94.4	6	85.7	
Hysterectomy^c^	1	6.6	1	14.3	
Smoking status					
Never	10	55.6	5	71.4	
Former	8	44.4	2	28.6	

Characteristics of participants from the RCT divided by genotype

^a^One participant was only 48 years, but had not had her menses for 6 years. All other participants were older than 50 years

^b^One participant had had a unilateral oophorectomy

^c^One participant had a combined hysterectomy and oophorectomy; and one participant had a hysterectomy before last menses, but was older than 60 years

^d^ *P*-values for comparison of baseline characteristics using Student’s t-test

## Discussion

In the present study, polymorphisms in *CYP19A1* were associated with circulating blood levels of female sex-hormones and there was interaction between genotypes and alcohol consumption in relation to blood levels. Specifically, *CYP19A1*/rs1062033 was associated to both estrone sulphate levels and to alcohol-dependent differences in estrone sulphate levels. *CYP19A1*/rs3751591 was both associated with SHBG levels and with risk of BC such that homozygous variant allele carriers had increased levels of serum SHBG and were at increased risk of BC. In addition we found indications of interaction between NSAID use, *CYP19A1* polymorphisms and levels of circulating female sex-hormones. We found that *CYP19A1* polymorphisms interacted with polymorphisms in *PPARGC1A* in relation to risk of BC, but there was no interaction with alcohol intake. Additionally, we showed that acute alcohol consumption affected circulating blood hormone and SHBG levels, but Ibuprofen intake and *PPARG* Pro^12^Ala status did not affect the found sex-hormone concentrations.

In the prospective study, we were able to show that inherent variations in *CYP19A1* were associated with up to 24 % differences in female sex-hormone levels, and *CYP19A1*/rs3751591 was both associated with SHBG levels and with risk of BC. *CYP19A1*/rs3751591 and the GGG/GAG (*CYP19A1*/A-rs10046-G, A-rs6493487-G, A-rs10519297-G) haplotype combination were associated with BC risk, but these results were based on very low numbers of cases and should therefore be interpreted with caution. *CYP19A1*/rs3751591 has no known function; it was chosen as a tag SNP and is located in a recombination hot spot. However, our results are in agreement with several other studies showing association between *CYP19A1* polymorphisms and estrogens [53, 55, 56, 66], but not BC risk [67–69]. The results may indicate that the 10–20 % genetically determined variation in sex hormone levels contributes little to BC risk and thus, that other factors may contribute more to hormone-dependent BC risk. These factors could be hormone replacement therapy, child birth, and age of menarche and menopause.

We found no evidence of interaction between genetically determined variation in aromatase and PPARγ activity. When interaction with alcohol was included in the analysis, we found that only the *PPARG* Pro^12^Ala wild type carriers were at significantly increased risk of BC irrespectively of *CYP19A1* genotypes. This indicates that the effect of the *PPARG* Pro^12^Ala polymorphism is very strong and that the *CYP19A1* polymorphisms only have minor influence on BC risk. We observed a possibly interaction between aromatase and PGC-1α, however, none of the *PPARGC1A* polymorphisms interacted with both *CYP19A1* polymorphisms and alcohol. Almost all risk estimates were above unity indicating a strong effect of alcohol regardless of genotype combinations. We have previously found evidence of *PPARG* and *PPARGC1A* being involved in alcohol-related BC [30]; however, based on the present findings, we cannot extend this mechanism to include aromatase. This may indicate that PPARγ mediates alcohol-related BC by additional mechanisms in addition to the one involving effects on aromatase.

We used a nested case-control design for the prospective study, which together with complete follow-up minimizes selection bias. In addition, information on life style factors was collected at enrolment, which minimizes the risk for differential misclassification between cases and controls. The study is fairly large to study main effects, it is homogenous and alcohol consumption is relatively high in the DCH cohort [70] making it suitable for studying gene-environment interactions with alcohol. However, we are aware that there are several limitations in studying gene-environment interaction with NSAID use including the limited power. The information on NSAID use retrieved from the FFQ may not necessarily reflect a long-term chronic use which is considered necessary to confer an effect on carcinogenesis [71, 72]. Moreover, NSAID use included different types of pain killers such as paracetamol, aspirin and Ibuprofen, which have different pharmacological effects and also different effect in relation to BC risk [42]. The genes were carefully selected based on their role in steroidogenesis and alcohol-related BC. The *CYP19A1* polymorphisms were mainly tag SNPs, whereas the *PPARG* and *PPARGC1A* polymorphisms were functional. However, only the interaction between *CYP19A1*/rs4646 and *PPARGC1A* Thr^612^Met, and the effect of *CYP19A1*/rs749292 on estrone sulphate levels withstood correction for multiple analyses when taking the number of analysed SNPs into account (main effects). Therefore, some of the results based on the prospective study may be due to chance.

After acute ingestion of alcohol, estrone, estrone sulphate and SHBG levels declined significantly and correlated inversely with ethanol blood levels. Both controlled acute trials [16, 20–23, 73], controlled trials with fixed amounts of alcohol over longer periods [17, 18] and observational studies [12–15, 24, 25] have reported increased levels of estrogens after consumption of alcohol. Most acute studies have only measured estradiol levels, which have been consistently increased after consumption of alcohol [16, 19–23], whereas estrone has only been measured in two acute studies in women using oral contraceptives [23] and HRT [20], respectively. In the study by Sarkola et al., estradiol increased after acute alcohol administration, but alcohol intake had no effect on estrone levels. However, the estradiol-to-estrone ratio was significantly increased. In a study by Ginsburg et al., estrone declined after acute alcohol consumption, whereas estradiol increased. Long-term interventional and observational studies most consistently report either increased levels of estradiol [13, 15, 18, 25, 74–76] and/or estrone [12, 13, 18, 75, 76] and/or estrone sulphate [14, 17, 75] and decreased SHBG levels [12, 75–77] among alcohol drinkers in both pre- and postmenopausal women. However, estradiol and estrone have very short half-lives of 35 min, whereas estrone sulphate has a half-life of 5–7 h [17]. Therefore, only acute studies, where blood is collected immediately after alcohol ingestion, are able to correctly measure the effect of alcohol on estradiol and estrone blood levels. On the other hand, acute ingestion of alcohol may have different effects on sex-steroids than chronic alcohol consumption, as illustrated by studies on alcohol consumption and immune effects [78]. It has been suggested that acute ingestion of alcohol affects catabolism of the hormones in the liver rather than synthesis [22, 23, 79, 80]. In the liver, alcohol consumption increases the [NADH]: [NAD+] ratio which leads to a decreased catabolism of sex-hormones mediated by 17β-hydroxysteroid dehydrogenase type 2 enzyme resulting in increased levels of testosterone and estradiol and decreased levels of androstenedione and estrone. Furthermore, it has been shown that only long-term chronic ingestion of alcohol induces aromatase [22, 34]. We detected a decline in estrone sulphate levels shortly after ingestion of alcohol, supporting that acute alcohol intake affects metabolism of female sex-hormone, which may explain the discrepancies between results from observational and experimental studies.

The RCT also has several limitations. Our aim was to conduct a pilot study to examine whether the *PPARG* Pro^12^Ala polymorphisms had any influence on the blood hormone level after consumption of alcohol with and without concurrent intake of Ibuprofen. Based on a controlled long-term feeding study [17], we should have an 80 % chance of detecting a change in hormone levels of 10 % on a 5 % significance level (α = 0.05) with 11 participants in each group. However, we were only able to recruit 7 *PPARG*^12^Ala variant-carriers. Nevertheless, we found statistically significant decreases in estrone, estrone sulphate and SHBG levels. We did not include an alcohol placebo group because our main aim was to examine the effect of concurrent use of Ibuprofen and alcohol consumption on circulating hormone levels. Therefore, the hormone effect could potentially be an effect of the ingredients in the alcoholic drink. However, other interventional studies have used similar ingredients in the alcoholic test drink e.g. different types of fruit juices [17, 18, 20, 22, 23, 73, 81] or pure glucose [16] without an effect on hormone levels. In order to verify the results from the present study, a new study should preferably include a placebo group, and if feasible for ethical reasons should also assess effects over a longer exposure period. Moreover, other steroid hormones should be included to examine other effects of alcohol consumption on steroidogenesis and metabolism. The method used to measure hormones differs from all the other studies mentioned in this paper. We determined the hormones by LC-MS because of its documented specificity [82] and to avoid overestimation due to lack of specificity of antibodies, which is a well-known challenge with conventional radioimmunoassays [82, 83]. However, MS methods suffer from lack of sensitivity and, consequently, we were not able to include results on estradiol measurements and 11 % of the estrone measurements had levels below zero which further decreased the statistical power.

## Conclusion

Our results show that alcohol consumption and inherent variations in *CYP19A1* influence the level of circulating blood sex-hormones. Specifically, *CYP19A1*/rs1062033 was associated to both estrone sulphate levels and to alcohol-dependent differences in estrone sulphate levels. *CYP19A1*/rs3751591 was both associated with SHBG levels and with risk of BC such that homozygous variant allele carriers had increased levels of serum SHBG and were at increased risk of BC. However, the genetically determined differences in sex hormone levels were not convincingly associated with BC risk. The results therefore indicate that the genetically determined variation in sex-hormone levels contributes little to BC risk and thus, that other factors may contribute more to hormone-dependent BC risk. In addition, our results indicate that acute and chronic alcohol consumption may affect metabolism and biosynthesis of estrogens differently. We were unable to show that aromatase is part of the mechanism of PPARγ-dependent alcohol-related BC.

## Additional files

Additional file 1: LD plot of *CYP19A1* with illustration of rs3751591 (in green square) located between two large haplotype blocks (genotyping data set from Utah residents with ancestry from Northern and Western Europe (CEU, version 3, release 2) in combination with Haploview (version 4.2, Broad Institute, Cambridge USA). (DOCX 487 kb) Additional file 2: Plasma levels of estrone, estrone sulphate and SHBG among 335 never and past users of hormone replacement therapy as percentage difference in hormonal measurements in relation to CYP19A1 polymorphisms divided by usage of NSAID. (DOCX 25 kb) Additional file 3: Risk estimates for different combinations of CYP19A1 haplotypes in relation to risk of BC. (DOCX 12 kb) Additional file 4 IRR for BC in relation to CYP19A1 polymorphisms per increment of 10 g alcohol per day. (DOCX 22 kb) Additional file 5: IRR for BC in relation to use of NSAID and CYP19A1 polymorphisms. (DOCX 30 kb) Additional file 6: IRR for BC in relation to combinations of PPARG Pro12Ala and CYP19A1 genotypes. (DOCX 33 kb) Additional file 7: IRR for BC in relation to combinations of PPARGC1A Gly482Ser and CYP19A1 genotypes. (DOCX 30 kb) Additional file 8: IRR for BC in relation to combinations of PPARGC1A Thr612Met and CYP19A1 genotypes. (DOCX 28 kb) Additional file 9: IRR for BC per 10 g alcohol/day for combinations of PPARG Pro12Ala and CYP19A1 genotypes. (DOCX 30 kb) Additional file 10: IRR for BC per 10 g alcohol/day for combinations PPARGC1A Gly482Ser and CYP19A1 genotypes. (DOCX 29 kb) Additional file 11: IRR for BC per 10 g alcohol/day for combinations of PPARGC1A Thr612Met and CYP19A1 genotypes. (DOCX 28 kb) Additional file 12: IRR for BC per 10 g alcohol/day in relation to combinations of NSAID use and CYP19A1 polymorphisms. (DOCX 29 kb) Additional file 13: Hormones, SHBG and ethanol measurements after acute ingestion of alcohol from the RCT study. (DOCX 19 kb)

## Abbreviations

BC: Breast cancer
BMI: Body Mass Index
CI: Confidence interval
CV: Coefficient of variation
CYP19A1: Cytochrome P450, family 19, subfamily A, polypeptide 1: encodes aromatase
DCH: Diet, Cancer and Health
HRT: Hormone replacement therapy
IRR: Incidence rate ratios
LC-MS: Liquid-Chromatography Mass-Spectrometry
LOD: Limit of detection
NSAID: Non-steroidal anti-inflammatory drugs
PGC-1α: Peroxisome proliferator-activated receptor gamma coactivator 1-α, encoded by PPARGC1A
PPARγ: Peroxisome proliferator-activated receptor gamma, encoded by PPARG
RCT: Randomised controlled trial
RXR: Retinoid X receptor
SNP: Single nucleotide polymorphism
