# Supplementary material for: Alcohol-related breast cancer in postmenopausal women – effect of CYP19A1, PPARG and PPARGC1A polymorphisms on female sex-hormone levels and interaction with alcohol consumption and NSAID usage in a nested case-control study and a randomised controlled trial
Source: BMC Cancer. 2016 Apr 21;16:283. doi: 10.1186/s12885-016-2317-y (PMC4839098; doi:10.1186/s12885-016-2317-y)

Additional file 1. LD plot of *CYP19A1* with illustration of rs3751591 (in green square) located between two large haplotype blocks (genotyping data set from Utah residents with ancestry from Northern and Western Europe (CEU, version 3, release 2) in combination with Haploview (version 4.2, Broad Institute, Cambridge USA).


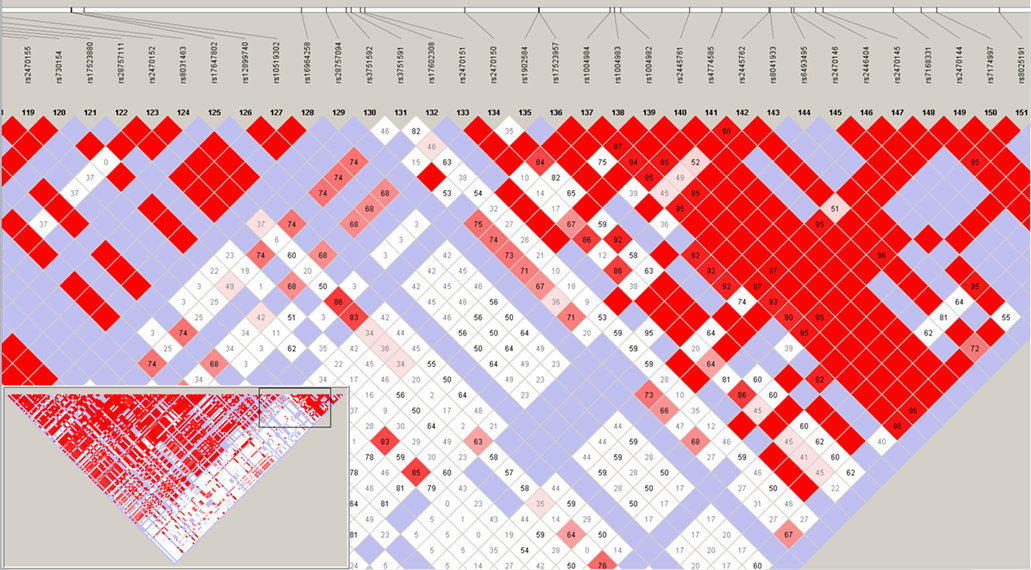

Supplement: Additional file 1: — LD plot of CYP19A1 with illustration of rs3751591 (in green square) located between two large haplotype blocks (genotyping data set from Utah residents with ancestry from Northern and Western Europe (CEU, version 3, release 2) in combination with Haploview (version 4.2, Broad Institute, Cambridge USA). (DOCX 487 kb) [file 12885_2016_2317_MOESM1_ESM.docx]
