# Supplementary material for: Alcohol-related breast cancer in postmenopausal women – effect of CYP19A1, PPARG and PPARGC1A polymorphisms on female sex-hormone levels and interaction with alcohol consumption and NSAID usage in a nested case-control study and a randomised controlled trial
Source: BMC Cancer. 2016 Apr 21;16:283. doi: 10.1186/s12885-016-2317-y (PMC4839098; doi:10.1186/s12885-016-2317-y)
Supplement: Additional file 2: — Plasma levels of estrone, estrone sulphate and SHBG among 335 never and past users of hormone replacement therapy as percentage difference in hormonal measurements in relation to CYP19A1 polymorphisms divided by usage of NSAID. (DOCX 25 kb) [file 12885_2016_2317_MOESM2_ESM.docx]

**Additional file 2: Plasma levels of estrone, estrone sulphate and SHBG among 335 never and past users of hormone replacement therapy as percentage change in hormonal measurements in relation to CYP19A1 polymorphisms divided by usage of NSAID.**

| Genotype | No  n (%)  n=217 | Yes  n (%)  n=118 | Estrone | | P-value | Estrone sulphate | | P-value | SHBG | | P-value |
| --- | --- | --- | --- | --- | --- | --- | --- | --- | --- | --- | --- |
|  |  |  | No | Yes |  | No | Yes |  | No | Yes |  |
|  |  |  | ∆ (95% CI)^a^ | ∆ (95% CI)^a^ |  | ∆ (95% CI)^a^ | ∆ (95% CI)^a^ |  | ∆ (95% CI)^a^ | ∆ (95% CI)^a^ |  |
| rs10519297  AA  AG+GG | 51 (24)  166 (76) | 28 (24)  90 (76) | 0 (ref.)  3 (-10;19) | -14 (-30;6)  -8 (-21;7) | 0.84 | 0 (ref.)  11 (-1;25) | -11 (-25;6)  -1 (-13;12) | 0.94 | 0 (ref.)  -8 (-19;5) | -3 (-20;17)  2 (-12;17) | 0.23 |
| rs749292  GG  AG+AA | 68 (31)  149 (69) | 33 (28)  85 (72) | 0 (ref.)  -5 (-16;8) | -14 (-28;4)  -16 (-27;-3) | 0.79 | 0 (ref.)  -15 (-24;-6) | -18 (-30;-5)  -22 (-30;-12) | 0.18 | 0 (ref.)  0 (-11;12) | 6 (-11;25)  8 (-5;22) | 0.87 |
| rs1062033  CC  CG+GG | 58 (27)  159 (73) | 30 (25)  88 (75) | 0 (ref.)  -3 (-15;10) | -13 (-28;6)  -15 (-26;-1) | 0.92 | 0 (ref.)  -14 (-23;-4) | -16 (-29;-1)  -22 (-31;-11) | 0.43 | 0 (ref.)  3 (-9;17) | 13 (-5;35)  8 (-5;24) | 0.47 |
| rs10046  AA  AG+GG | 54 (25)  163 (75) | 37 (31)  81 (69) | 0 (ref.)  -1 (-14;14) | -11 (-26;7)  -13 (-25;1) | 0.89 | 0 (ref.)  7 (-5;21) | -7 (-21;10)  -6 (-18;7) | 0.50 | 0 (ref.)  -3 (-15;10) | 1 (-15;20)  6 (-8;22) | 0.45 |
| rs4646  CC  CA+AA | 119 (55)  98 (45) | 64 (54)  54 (46) | 0 (ref.)  1 (-10;14) | -11 (-22;2)  -12 (-24;1) | 0.83 | 0 (ref.)  7 (-3;19) | -9 (-19;2)  -7 (-18;5) | 0.57 | 0 (ref.)  -6 (-15;5) | 4 (-8;18)  4 (-9;18) | 0.57 |
| rs6493487  AA  GA+GG | 121 (56)  96 (44) | 80 (68)  38 (32) | 0 (ref.)  -9 (-19;2) | -14 (-24;-3)  -19 (-31;-5) | 0.74 | 0 (ref.)  4 (-6;16) | -9 (-18;1)  -10 (-22;3) | 0.55 | 0 (ref.)  -10 (-19;0) | -1 (-11;11)  8 (-7;25) | 0.05 |
| rs2008691  AA  GA+GG | 148 (68)  69 (32) | 68 (58)  50 (42) | 0 (ref.)  11 (-2;26) | -9 (-20;3)  -9 (-21;5) | 0.31 | 0 (ref.)  2 (-8;14) | -13 (-22;2)  -8 (-18;4) | 0.73 | 0 (ref.)  1 (-10;14) | 4 (-8;17)  12 (-1;28) | 0.49 |
| rs3751591  TT+TC  CC | 213 (98)  4 (2) | 114 (97)  4 (3) | 0 (ref.)  10 (-29;71) | -13 (-21;-3)  15 (-26;77) | 0.59 | 0 (ref.)  -16 (-42;21) | -13 (-20;-5)  48 (3;114) | 0.008 | 0 (ref.)  71 (15;154) | 8 (-2;18)  17 (-21;74) | 0.12 |
| rs2445762  TT  TC+CC | 118 (54)  99 (46) | 54 (46)  64 (54) | 0 (ref.)  -3 (-14;9) | -12 (-23;2)  -14 (-25;-2) | 0.98 | 0 (ref.)  -1 (-10;10) | -10 (-20;2)  -13 (-22;-2) | 0.75 | 0 (ref.)  -4 (-14;7) | 10 (-4;25)  2 (-10;15) | 0.69 |
| rs11070844  CC  TC+TT | 173 (80)  44 (20) | 92 (78)  26 (22) | 0 (ref.)  14 (-1;31) | -15 (-24;-5)  13 (-5;35) | 0.20 | 0 (ref.)  16 (3;32) | -11 (-19;-2)  2 (-12;19) | 0.93 | 0 (ref.)  8 (-6;23) | 8 (-3;19)  11 (-6;31) | 0.69 |

Δ Percentage change in hormonal measurements compared to WT divided by usage of NSAID.

^a^ Adjusted for age, smoking (never, past, current), abstainers, alcohol intake (increment of 10 g per day) and BMI (kg/m^2^) at baseline.

^b^ P-value for interaction.

SHBG: Sex-hormone binding globulin.
