# Supplementary material for: Alcohol-related breast cancer in postmenopausal women – effect of CYP19A1, PPARG and PPARGC1A polymorphisms on female sex-hormone levels and interaction with alcohol consumption and NSAID usage in a nested case-control study and a randomised controlled trial
Source: BMC Cancer. 2016 Apr 21;16:283. doi: 10.1186/s12885-016-2317-y (PMC4839098; doi:10.1186/s12885-016-2317-y)
Supplement: Additional file 3: — Risk estimates for different combinations of CYP19A1 haplotypes in relation to risk of BC. (DOCX 12 kb) [file 12885_2016_2317_MOESM3_ESM.docx]

**Additional file 3: Risk estimates for different combinations of *CYP19A1* haplotypes in relation to risk of BC.**

| Haplotype^a^ | **AAA** | G**A**G | GGG |
| --- | --- | --- | --- |
| **AAA** | 1.00 (ref.) (164/162) | 0.91 (0.68-1.22) (164/182) | 1.17 (0.86-1.59) (156/143) |
| G**A**G |  | 1.25 (0.79-1.97) (48/43) | 1.56 (1.02-2.40) (74/56) |
| GGG |  |  | 0.99 (0.59-1.68) (34/35) |

IRR (95% CI) for BC for different combinations of haplotypes. The number of cases and controls in each cell is listed. Variant alleles are bold. Adjusted for parous/nulliparous, number of births, age at first birth, length of school education (low, medium, high), duration of HRT use (years), BMI (kg/m^2^) and alcohol intake (10 g/day).

^a^Haplotype sequence: rs10046, rs6493487, rs10519297
