# Supplementary material for: Alcohol-related breast cancer in postmenopausal women – effect of CYP19A1, PPARG and PPARGC1A polymorphisms on female sex-hormone levels and interaction with alcohol consumption and NSAID usage in a nested case-control study and a randomised controlled trial
Source: BMC Cancer. 2016 Apr 21;16:283. doi: 10.1186/s12885-016-2317-y (PMC4839098; doi:10.1186/s12885-016-2317-y)
Supplement: Additional file 4 — IRR for BC in relation to CYP19A1 polymorphisms per increment of 10 g alcohol per day. (DOCX 22 kb) [file 12885_2016_2317_MOESM4_ESM.docx]

**Additional file 4: IRR for BC in relation to *CYP19A1* polymorphisms per increment of 10 g alcohol per day.**

| Genotype | n_cases_ (%)  (n=651) | n_controls_ (%)  (n=651) | IRR^a^ (95% CI) | IRR^b^ (95% CI) | P-value^c^ |
| --- | --- | --- | --- | --- | --- |
| rs10519297  AA  AG+GG | 159 (24)  492 (76) | 162 (25)  489 (75) | 1.13 (0.96-1.33)  1.16 (1.06-1.27) | 1.12 (0.94-1.32)  1.14 (1.05-1.25) | 0.79 |
| rs749292  GG  AG+AA | 208 (32)  443 (68) | 193 (30)  458 (70) | 1.23 (1.07-1.42)  1.12 (1.02-1.23) | 1.23 (1.07-1.43)  1.10 (1.00-1.21) | 0.18 |
| rs1062033  CC  CG+GG | 196 (30)  455 (70) | 177 (27)  474 (73) | 1.24 (1.07-1.43)  1.13 (1.03-1.23) | 1.23 (1.06-1.43)  1.10 (1.01-1.21) | 0.22 |
| rs10046  AA  AG+GG | 171 (26)  480 (74) | 176 (27)  475 (73) | 1.16 (0.73-1.48)  1.15 (1.06-1.26) | 1.14 (0.96-1.34)  1.14 (1.04-1.24) | 0.98 |
| rs4646  CC  CA+AA | 355 (55)  296 (45) | 349 (54)  302 (46) | 1.14 (1.03-1.27)  1.17 (1.04-1.31) | 1.18 (1.00-1.24)  1.16 (1.04-1.30) | 0.62 |
| rs6493487  AA  GA+GG | 384 (59)  267 (41) | 405 (62)  246 (38) | 1.13 (1.02-1.25)  1.18 (1.04-1.33) | 1.12 (1.01-1.24)  1.16 (1.03-1.31) | 0.64 |
| rs2008691  AA  GA+GG | 453 (70)  198 (30) | 443 (68)  208 (32) | 1.13 (1.03-1.23)  1.22 (1.05-1.41) | 1.11 (1.01-1.22)  1.20 (1.04-1.38) | 0.37 |
| rs3751591  TT+TC  CC | 626 (96)  25 (4) | 638 (98)  13 (2) | 1.15 (1.06-1.24)  1.66 (0.86-3.18) | 1.13 (1.04-1.22)  1.66 (0.88-3.14) | 0.24 |
| rs2445762  TT  TC+CC | 339 (52)  312 (48) | 347 (53)  304 (47) | 1.13 (1.02-1.25)  1.18 (1.06-1.32) | 1.11 (1.00-1.23)  1.18 (1.05-1.32) | 0.43 |
| rs11070844  CC  TC+TT | 520 (80)  131 (20) | 526 (81)  125 (19) | 1.17 (1.07-1.27)  1.11 (0.95-1.30) | 1.18 (1.05-1.25)  1.11 (0.95-1.29) | 0.69 |

^a^Crude.

^b^Adjusted for parity (parous/nulliparous, number of births, age at first birth), length of school education (low, medium, high), duration of HRT use (years) and body mass index (kg/m2) at baseline.

^c^P-value for interaction for adjusted risk estimates.
