# Supplementary material for: Alcohol-related breast cancer in postmenopausal women – effect of CYP19A1, PPARG and PPARGC1A polymorphisms on female sex-hormone levels and interaction with alcohol consumption and NSAID usage in a nested case-control study and a randomised controlled trial
Source: BMC Cancer. 2016 Apr 21;16:283. doi: 10.1186/s12885-016-2317-y (PMC4839098; doi:10.1186/s12885-016-2317-y)
Supplement: Additional file 5: — IRR for BC in relation to use of NSAID and CYP19A1 polymorphisms. (DOCX 30 kb) [file 12885_2016_2317_MOESM5_ESM.docx]

**Additional file 5. IRR for BC in relation to use of NSAID and *CYP19A1* polymorphisms.**

| Genotype | No  n_cases_/  n_controls_  (n=675) | Yes  n_cases_/  n_controls_  (n=675) | No  IRR (95% CI)^a^ | Yes  IRR (95% CI)^a^ | No  IRR (95% CI)^b^ | Yes  IRR (95% CI)^b^ | *P*-value^c^ |
| --- | --- | --- | --- | --- | --- | --- | --- |
| rs10519297  AA  AG+GG | 87/111  305/358 | 81/59  202/177 | 1.00 (ref.)  1.21 (0.87-1.68) | 1.69 (1.08-2.63)  1.45 (1.03-2.05) | 1.00 (ref.)  1.16 (0.82-1.63) | 1.65 (1.05-2.59)  1.44 (1.00-2.05) | 0.30 |
| rs749292  GG  AG+AA | 122/134  270/305 | 91/67  192/169 | 1.00 (ref.)  0.96 (0.71-1.31) | 1.44 (0.98-2.12)  1.21 (0.88-1.67) | 1.00 (ref.)  1.00 (0.73-1.36) | 1.53 (1.03-2.28)  1.26 (0.90-1.75) | 0.42 |
| rs1062033  CC  CG+GG | 114/120  278/319 | 86/62  197/174 | 1.00 (ref.)  0.89 (0.65-1.22) | 1.40 (0.94-2.10)   - 1. (0.82-1.59) | 1.0 (ref.)  0.92 (0.67-1.26) | 1.48 (0.98-2.23)  1.18 (0.84-1.65) | 0.58 |
| rs10046  AA  AG+GG | 93/120  299/319 | 86/63  197/173 | 1.00 (ref.)  1.22 (0.89-1.68) | 1.69 (1.11-2.58)  1.46 (1.04-2.04) | 1.00 (ref.)  1.17 (0.84-1.61) | 1.68 (1.09-2.59)  1.43 (1.01-2.02) | 0.24 |
| rs4646  CC  CA+AA | 204/230  188/209 | 163/132  120/104 | 1.00 (ref.)  1.01 (0.76-1.32) | 1.32 (0.99-1.76)  1.30 (0.94-1.80) | 1.00 (ref.)  0.97 (0.73-1.28) | 1.33 (0.99-1.78)  1.30 (0.94-1.82) | 0.95 |
| rs6493487  AA  GA+GG | 223/277  169/162 | 177/146  106/90 | 1.00 (ref.)  1.31 (1.00-1.73) | 1.46 (1.11-1.92)  1.46 (1.05-2.03) | 1.00 (ref.)  1.30 (0.98-1.73) | 1.49 (1.12-1.97)  1.47 (1.05-2.08) | 0.26 |
| rs2008691  AA  GA+GG | 273/297  119/142 | 197/164  86/72 | 1.00 (ref.)  0.89 (0.66-1.19) | 1.26 (0.96-1.64)  1.27 (0.90-1.79) | 1.00 (ref.)  0.88 (0.65-1.19) | 1.27 (0.96-1.67)  1.31 (0.92-1.87) | 0.51 |
| rs3751591  TT+TC  CC | 374/429  18/10 | 275/233  8/3 | 1.00 (ref.)  2.14 (0.93-4.91) | 1.32 (1.06-1.65)  3.04 (0.80-11.60) | 1.00 (ref.)  2.09 (0.90-4.88) | 1.35 (1.08-1.69)  3.37 (0.85-13.36) | 0.83 |
| rs2445762  TT  TC+CC | 207/228  185/211 | 147/128  136/108 | 1.00 (ref.)  0.96 (0.73-1.27) | 1.24 (0.92-1.67)   - 1. 0.98-1.84) | 1.0 (ref.)  0.97 (0.73-1.29) | 1.24 (0.91-1.68)  1.43 (1.03-1.97) | 0.48 |
| rs11070844  CC  TC+TT | 325/357  67/82 | 216/192  67/44 | 1. (ref.)   0.91 (0.64-1.31) | 1.21 (0.95-1.54)  1.62 (1.09-2.41) | 1.00 (ref.)  0.94 (0.65-1.36) | 1.26 (0.98-1.61)  1.59 (1.05-2.39) | 0.30 |

^a^Crude.

^b^Adjusted for parity (parous/nulliparous, number of births, age at first birth), length of school education (low, medium, high), duration of HRT use (years), body mass index (kg/m^2^), and alcohol intake (increment of 10 g per day) at baseline.

^c^*P*-value for interaction for adjusted risk estimates.
