# Supplementary material for: Alcohol-related breast cancer in postmenopausal women – effect of CYP19A1, PPARG and PPARGC1A polymorphisms on female sex-hormone levels and interaction with alcohol consumption and NSAID usage in a nested case-control study and a randomised controlled trial
Source: BMC Cancer. 2016 Apr 21;16:283. doi: 10.1186/s12885-016-2317-y (PMC4839098; doi:10.1186/s12885-016-2317-y)
Supplement: Additional file 6: — IRR for BC in relation to combinations of PPARG Pro12Ala and CYP19A1 genotypes. (DOCX 33 kb) [file 12885_2016_2317_MOESM6_ESM.docx]

**Additional file 6: IRR for BC in relation to combinations of *PPARG* Pro^12^Ala and *CYP19A1* genotypes**

| Genotype | PPARG Pro^12^Ala | | PPARG Pro^12^Ala | | PPARG Pro^12^Ala | | P-value^c^ |
| --- | --- | --- | --- | --- | --- | --- | --- |
|  | Pro/Pro  n_cases_/ n_controls_  (n=685) | Ala-carriers  n_case_/ n_controls_  (n=685) | Pro/Pro  IRR (95% CI)^a^ | Ala-carriers  IRR (95% CI) ^a^ | Pro/Pro  IRR (95% CI)^b^ | Ala-carriers  IRR (95% CI)^b^ |  |
| rs10519297  AA  AG+GG | 132/121  396/367 | 37/51  120/146 | 1. (ref.)   0.99 (0.75-1.31) | 0.65 (0.39-1.08)  0.75 (0.52-1.08) | 1. (ref.)   0.95 (0.71-1.27) | 0.61 (0.36-1.03)  0.75 (0.52-1.09) | 0.38 |
| rs749292  GG  AG+AA | 165/143  363/345 | 51/60  106/137 | 1. (ref.)    1. 0.68-1.21) | 0.73 (0.46-1.14)  0.67 (0.47-0.94) | 1.00 (ref.)  0.92 (0.68-1.23) | 0.75 (0.47-1.18)  0.68 (0.48-0.98) | 1.00 |
| rs1062033  CC  CG+GG | 152/132  376/356 | 51/54  106/143 | 1.00 (ref.)  0.90 (0.67-1.21) | 0.81 (0.51-1.27)  0.63 (0.45-0.91) | 1.00 (ref.)  0.91 (0.68-1.23) | 0.84 (0.52-1.34)  0.65 (0.45-0.93) | 0.57 |
| rs10046  AA  AG+GG | 137/131  391/357 | 44/55  113/142 | 1.00 (ref.)  1.04 (0.79-1.36) | 0.76 (0.47-1.21)  0.75 (0.53-1.08) | 1.00 (ref.)  1.00 (0.76-1.32) | 0.75 (0.47-1.22)  0.75 (0.51-1.08) | 0.97 |
| rs4646  CC  CA+AA | 286/252  242/236 | 84/117  73/80 | 1.00 (ref.)  0.90 (0.71-1.14) | 0.62 (0.44-0.87)  0.80 (0.56-1.16) | 1.00 (ref.)  0.88 (0.69-1.13) | 0.63 (0.44-0.90)  0.80 (0.55-1.15) | 0.17 |
| rs6493487  AA  GA+GG | 306/302  222/186 | 99/126  58/71 | 1.00 (ref.)  1.20 (0.93-1.54) | 0.78 (0.57-1.06)  0.82 (0.54-1.24) | 1.00 (ref.)  1.18 (0.91-1.53) | 0.78 (0.57-1.08)  0.84 (0.55-1.29) | 0.74 |
| rs2008691  AA  GA+GG | 372/334  156/154 | 106/134  51/63 | 1.00 (ref.)  0.89 (0.69-1.17) | 0.70 (0.52-0.96)  0.71 (0.47-1.07) | 1.00 (ref.)  0.89 (0.67-1.17) | 0.71 (0.52-0.97)  0.74 (0.49-1.13) | 0.53 |
| rs3751591  TT+TC  CC | 508/478  20/10 | 151/194  6/3 | 1.00 (ref.)  1.83 (0.82-4.10) | 0.74 (0.57-0.95)  2.11 (0.41-10.97) | 1.00 (ref.)  1.68 (0.74-3.81) | 0.75 (0.57-0.97)  2.88 (0.54-15.33) | 0.37 |
| rs2445762  TT  TC+CC | 271/260  257/228 | 87/104  70/93 | 1.00 (ref.)  1.07 (0.84-1.36) | 0.79 (0.56-1.12)  0.71 (0.50-1.03) | 1.00 (ref.)  1.11 (0.87-1.43) | 0.83 (0.59-1.18)  0.74 (0.51-1.07) | 0.37 |
| rs11070844  CC  TC+TT | 435/396  93/92 | 116/159  41/38 | 1.00 (ref.)  0.94 (0.69-1.29) | 0.66 (0.50-0.88)  0.99 (0.62-1.58) | 1.00 (ref.)   - 1. (0.70-1.33) | 0.68 (0.52-0.92)  0.96 (0.59-1.55) | 0.24 |

^a^Crude.

^b^Adjusted for parity (parous/nulliparous, number of births, age at first birth), length of school education (low, medium, high), duration of HRT use (years), body mass index (kg/m2) and alcohol intake (increment of 10 g per day) at baseline.

^c^P-value for interaction for the adjusted risk estimates.
