# Supplementary material for: Alcohol-related breast cancer in postmenopausal women – effect of CYP19A1, PPARG and PPARGC1A polymorphisms on female sex-hormone levels and interaction with alcohol consumption and NSAID usage in a nested case-control study and a randomised controlled trial
Source: BMC Cancer. 2016 Apr 21;16:283. doi: 10.1186/s12885-016-2317-y (PMC4839098; doi:10.1186/s12885-016-2317-y)
Supplement: Additional file 7: — IRR for BC in relation to combinations of PPARGC1A Gly482Ser and CYP19A1 genotypes. (DOCX 30 kb) [file 12885_2016_2317_MOESM7_ESM.docx]

**Additional file 7: IRR for BC in relation to combinations of *PPARGC1A* Gly^482^Ser and *CYP19A1* genotypes**

| Genotype | PPARGC1A Gly^482^Ser | | PPARGC1A Gly^482^Ser | | PPARGC1A Gly^482^Ser | | P-value^c^ |
| --- | --- | --- | --- | --- | --- | --- | --- |
|  | Gly-carriers  n_cases_/ n_controls_  (n=686) | Ser/Ser  n_case_/ n_controls_  (n=686) | Gly-carriers  IRR (95% CI)^a^ | Ser/Ser  IRR (95% CI) ^a^ | Gly-carriers  IRR (95% CI)^b^ | Ser/Ser  IRR (95% CI)^b^ |  |
| rs10519297  AA  AG+GG | 147/148  461/449 | 21/26  55/63 | 1.00 (ref.)  1.04 (0.80-1.36) | 0.83 (0.45-1.52)  0.89 (0.57-1.37) | 1.00 (ref.)  1.02 (0.77-1.34) | 0.78 (0.42-1.46)  0.83 (0.53-1.29) | 0.92 |
| rs749292  GG  AG+AA | 192/181  418/416 | 24/22  52/67 | 1.00 (ref.)  0.94 (0.73-1.21) | 1.10 (0.57-2.12)  0.72 (0.47-1.11) | 1.00 (ref.)  0.95 (0.73-1.23) | 1.02 (0.52-2.00)  0.69 (0.44-1.08) | 0.41 |
| rs1062033  CC  CG+GG | 179/166  431/431 | 24/20  52/69 | 1.00 (ref.)  0.91 (0.71-1.17) | 1.22 (0.62-2.39)  0.67 (0.43-1.04) | 1.00 (ref.)  0.91 (0.70-1.18) | 1.14 (0.57-2.26)  0.64 (0.41-1.01) | 0.24 |
| rs10046  AA  AG+GG | 162/164  448/433 | 20/24  56/65 | 1.00 (ref.)  1.07 (0.83-1.37) | 0.86 (0.46-1.62)  0.89 (0.58-1.36) | 1.00 (ref.)  1.03 (0.80-1.34) | 0.84 (0.44-1.60)  0.81 (0.52-1.26) | 0.88 |
| rs4646  CC  CA+AA | 326/326  284/271 | 45/45  31/44 | 1.00 (ref.)  1.06 (0.84-1.32) | 1.00 (0.64-1.55)  0.72 (0.44-1.16) | 1.00 (ref.)  1.04 (0.82-1.31) | 0.98 (0.62-1.54)  0.65 (0.40-1.07) | 0.21 |
| rs6493487  AA  GA+GG | 364/369  246/228 | 43/60  33/29 | 1.00 (ref.)  1.12 (0.89-1.42) | 0.74 (0.49-1.13)  1.24 (0.71-2.18) | 1.00 (ref.)  1.10 (0.86-1.40) | 0.68 (0.44-1.05)  1.23 (0.69-2.19) | 0.19 |
| rs2008691  AA  GA+GG | 427/406  183/191 | 51/63  25/26 | 1.00 (ref.)  0.91 (0.71-1.15) | 0.78 (0.52-1.16)  0.92 (0.52-1.62) | 1.00 (ref.)  0.91 (0.71-1.17) | 0.73 (0.48-1.11)  0.89 (0.50-1.60) | 0.43 |
| rs3751591  TT+TC  CC | 584/586  25/9 | 76/87  -/2 | 1.00 (ref.)  2.50 (1.15-5.42) | 0.91 (0.65-1.27)  - | 1.00 (ref.)  2.52 (1.15-5.56) | 0.87 (0.61-1.23)  - | 0.02 |
| rs2445762  TT  TC+CC | 322/319  288/278 | 36/46  40/43 | 1.00 (ref.)  1.02 (0.81-1.29) | 0.79 (0.49-1.27)  0.91 (0.58-1.44) | 1.00 (ref.)  1.05 (0.83-1.34) | 0.76 (0.47-1.23)  0.89 (0.56-1.41) | 0.76 |
| rs11070844  CC  TC+TT | 493/480  117/117 | 59/75  17/14 | 1.00 (ref.)  0.99 (0.75-1.31) | 0.77 (0.53-1.11)  1.26 (0.61-2.61) | 1.00 (ref.)  0.99 (0.74-1.32) | 0.74 (0.50-1.07)  1.16 (0.55-2.44) | 0.29 |

^a^Crude.

^b^Adjusted for parity (parous/nulliparous, number of births, age at first birth), length of school education (low, medium, high), duration of HRT use (years), body mass index (kg/m2) and alcohol intake (increment of 10 g per day) at baseline.

^c^P-value for interaction for the adjusted risk estimates.
