# Supplementary material for: Alcohol-related breast cancer in postmenopausal women – effect of CYP19A1, PPARG and PPARGC1A polymorphisms on female sex-hormone levels and interaction with alcohol consumption and NSAID usage in a nested case-control study and a randomised controlled trial
Source: BMC Cancer. 2016 Apr 21;16:283. doi: 10.1186/s12885-016-2317-y (PMC4839098; doi:10.1186/s12885-016-2317-y)
Supplement: Additional file 8: — IRR for BC in relation to combinations of PPARGC1A Thr612Met and CYP19A1 genotypes. (DOCX 28 kb) [file 12885_2016_2317_MOESM8_ESM.docx]

**Additional file 8: IRR for BC in relation to combinations of *PPARGC1A* Thr^612^Met and *CYP19A1* genotypes**

| Genotype | PPARGC1A Thr^612^Met | | PPARGC1A Thr^612^Met | | PPARGC1A Thr^612^Met | | P-value^c^ |
| --- | --- | --- | --- | --- | --- | --- | --- |
|  | Thr/Thr  n_cases_/ n_controls_  (n=686) | Met-carriers  n_case_/ n_controls_  (n=686) | Thr/Thr  IRR (95% CI)^a^ | Met-carriers  IRR (95% CI) ^a^ | Thr/Thr  IRR (95% CI)^b^ | Met-carriers  IRR (95% CI)^b^ |  |
| rs10519297  AA  AG+GG | 152/164  465/460 | 18/10  51/52 | 1.00 (ref.)  1.10 (0.85-1.42) | 2.12 (0.89-5.05)  1.08 (0.69-1.68) | 1.00 (ref.)  1.07 (0.82-1.39) | 1.87 (0.77-4.55)  1.02 (0.65-1.62) | 0.16 |
| rs749292  GG  AG+AA | 195/178  422/446 | 21/24  48/38 | 1.00 (ref.)  0.85 (0.66-1.09) | 0.77 (0.41-1.42)  1.20 (0.73-1.97) | 1.00 (ref.)  0.86 (0.67-1.12) | 0.77 (0.41-1.45)  1.13 (0.68-1.89) | 0.18 |
| rs1062033  CC  CG+GG | 186/163  431/461 | 17/22  52/40 | 1.00 (ref.)  0.81 (0.63-1.05) | 0.70 (0.36-1.34)  1.16 (0.71-1.91) | 1.00 (ref.)  0.82 (0.63-1.07) | 0.71 (0.36-1.38)  1.09 (0.65-1.81) | 0.13 |
| rs10046  AA  AG+GG | 163/177  454/447 | 19/11  50/51 | 1.00 (ref.)  1.12 (0.87-1.43) | 2.04 (0.89-4.67)  1.09 (0.70-1.70) | 1.00 (ref.)  1.07 (0.83-1.38) | 1.82 (0.78-4.26)  1.02 (0.65-1.61) | 0.63 |
| rs4646  CC  CA+AA | 326/347  291/277 | 45/24  24/38 | 1.00 (ref.)  1.11 (0.89-1.39) | 2.17 (1.25-3.76)  0.66 (0.38-1.12) | 1.00 (ref.)  1.09 (0.87-1.36) | 2.06 (1.17-3.65)  0.62 (0.36-1.08) | 0.002 |
| rs6493487  AA  GA+GG | 366/396  241/228 | 41/34  28/28 | 1.00 (ref.)  1.22 (0.97-1.54) | 1.29 (0.79-2.11)  1.16 (0.67-2.00) | 1.00 (ref.)  1.21 (0.95-1.53) | 1.20 (0.73-1.99)  1.12 (0.63-1.97) | 0.49 |
| rs2008691  AA  GA+GG | 427/428  190/196 | 51/41  18/21 | 1.00 (ref.)  0.96 (0.76-1.22) | 1.30 (0.83-2.04)  0.82 (0.43-1.57) | 1.00 (ref.)  0.97 (0.76-1.23) | 1.23 (0.77-1.95)  0.80 (0.41-1.57) | 0.35 |
| rs3751591  TT+TC  CC | 592/614  25/10 | 68/59  1/3 | 1.00 (ref.)  2.59 (1.20-5.59) | 1.20 (0.82-1.76)  - | 1.00 (ref.)  2.49 (1.14-5.45) | 1.14 (0.77-1.69)  - | 0.06 |
| rs2445762  TT  TC+CC | 320/331  297/293 | 38/33  31/29 | 1.00 (ref.)  1.05 (0.84-1.31) | 1.22 (0.75-1.99)  1.09 (0.63-1.89) | 1.00 (ref.)  1.08 (0.86-1.36) | 1.17 (0.70-1.94)  1.06 (0.60-1.87) | 0.64 |
| rs11070844  CC  TC+TT | 498/504  119/120 | 53/51  16/11 | 1.00 (ref.)  1.02 (0.77-1.35) | 1.06 (0.70-1.62)  1.47 (0.68-3.17) | 1.00 (ref.)  1.02 (0.76-1.36) | 1.02 (0.66-1.58)  1.36 (0.61-3.02) | 0.58 |

^a^Crude.

^b^Adjusted for parity (parous/nulliparous, number of births, age at first birth), length of school education (low, medium, high), duration of HRT use (years), body mass index (kg/m2) and alcohol intake (increment of 10 g per day) at baseline.

^c^P-value for interaction for the adjusted risk estimates.
