# Supplementary material for: Alcohol-related breast cancer in postmenopausal women – effect of CYP19A1, PPARG and PPARGC1A polymorphisms on female sex-hormone levels and interaction with alcohol consumption and NSAID usage in a nested case-control study and a randomised controlled trial
Source: BMC Cancer. 2016 Apr 21;16:283. doi: 10.1186/s12885-016-2317-y (PMC4839098; doi:10.1186/s12885-016-2317-y)
Supplement: Additional file 9: — IRR for BC per 10 g alcohol/day for combinations of PPARG Pro12Ala and CYP19A1 genotypes. (DOCX 30 kb) [file 12885_2016_2317_MOESM9_ESM.docx]

**Additional file 9: IRR for BC per 10 g alcohol/day for combinations of *PPARG* Pro^12^Ala and *CYP19A1* genotypes**

| Genotype | PPARG Pro^12^Ala | | PPARG Pro^12^Ala | | PPARG Pro^12^Ala | | P-value^c^ |
| --- | --- | --- | --- | --- | --- | --- | --- |
|  | Pro/Pro  n_cases_/ n_controls_  (n=650) | Ala-carriers  n_case_/ n_controls_  (n=650) | Pro/Pro  IRR (95% CI)^a^ | Ala-carriers  IRR (95% CI) ^a^ | Pro/Pro  IRR (95% CI)^b^ | Ala-carriers  IRR (95% CI)^b^ |  |
| rs10519297  AA  AG+GG | 128/115  375/350 | 31/46  116/139 | 1.17 (0.96-1.42)  1.24 (1.11-1.38) | 1.06 (0.77-1.46)  0.97 (0.82-1.14) | 1.15 (0.94-1.41)  1.23 (1.10-1.36) | 1.06 (0.77-1.47)  0.95 (0.81-1.12) | 0.08 |
| rs749292  GG  AG+AA | 158/136  345/329 | 50/57  97/128 | 1.39 (1.15-1.67)  1.16 (1.04-1.29) | 0.98 (0.77-1.23)  0.99 (0.83-1.19) | 1.39 (1.15-1.68)  1.14 (1.02-1.27) | 0.98 (0.77-1.24)  0.97 (0.80-1.17) | 0.03 |
| rs1062033  CC  CG+GG | 146/126  357/339 | 50/51  97/134 | 1.37 (1.13-1.66)  1.17 (1.05-1.30) | 1.01 (0.80-1.29)  0.96 (0.80-1.16) | 1.37 (1.13-1.66)  1.15 (1.03-1.29) | 1.01 (0.79-1.28)  0.95 (0.78-1.14) | 0.04 |
| rs10046  AA  AG+GG | 133/125  370/340 | 38/50  109/135 | 1.18 (0.98-1.41)  1.24 (1.11-1.37) | 1.12 (0.79-1.58)  0.96 (0.81-1.12) | 1.15 (0.95-1.39)  1.22 (1.10-1.36) | 1.12 (0.79-1.58)  0.94 (0.80-1.11) | 0.07 |
| rs4646  CC  CA+AA | 276/240  227/225 | 78/108  69/77 | 1.20 (1.06-1.35)  1.25 (1.09-1.43) | 0.95 (0.77-1.18)  1.00 (0.82-1.22) | 1.18 (1.04-1.33)  1.24 (1.08-1.43) | 0.93 (0.74-1.16)  1.00 (0.82-1.22) | 0.08 |
| rs6493487  AA  GA+GG | 293/287  210/178 | 90/117  57/68 | 1.18 (1.04-1.34)  1.27 (1.10-1.48) | 1.02 (0.84-1.23)  0.94 (0.76-1.18) | 1.16 (1.03-1.32)  1.26 (1.08-1.46) | 1.01 (0.83-1.22)  0.93 (0.74-1.16) | 0.07 |
| rs2008691  AA  GA+GG | 355/318  148/147 | 98/124  49/61 | 1.21 (1.08-1.34)  1.25 (1.05-1.50) | 0.90 (0.75-1.09)  1.12 (0.88-1.43) | 1.19 (1.07-1.33)  1.23 (1.03-1.47) | 0.89 (0.73-1.07)  1.11 (0.87-1.41) | 0.04 |
| rs3751591  TT+TC  CC | 483/455  20/10 | 142/182  5/3 | 1.21 (1.11-1.33)  1.66 (0.84-3.29) | 0.99 (0.85-1.14)  2.24 (0.27-18.71) | 1.20 (1.09-1.32)  1.71 (0.88-3.34) | 0.98 (0.84-1.13)  2.18 (0.25-19.35) | 0.07 |
| rs2445762  TT  TC+CC | 256/246  247/219 | 82/100  65/85 | 1.20 (1.06-1.36)  1.25 (1.09-1.43) | 0.97 (0.80-1.18)  1.01 (0.81-1.26) | 1.17 (1.03-1.33)  1.25 (1.09-1.43) | 0.96 (0.79-1.17)  0.99 (0.79-1.24) | 0.09 |
| rs11070844  CC  TC+TT | 411/378  92/87 | 109/148  38/37 | 1.23 (1.11-1.36)  1.17 (0.96-1.42) | 0.99 (0.84-1.16)  0.96 (0.71-1.30) | 1.22 (1.09-1.35)  1.16 (0.95-1.41) | 0.97 (0.82-1.15)  0.97 (0.71-1.31) | 0.10 |

^a^Crude.

^b^Adjusted for parity (parous/nulliparous, number of births, age at first birth), length of school education (low, medium, high), duration of HRT use (years) and body mass index (kg/m2).

^c^P-value for comparison of the adjusted risk estimates.
