# Supplementary material for: Alcohol-related breast cancer in postmenopausal women – effect of CYP19A1, PPARG and PPARGC1A polymorphisms on female sex-hormone levels and interaction with alcohol consumption and NSAID usage in a nested case-control study and a randomised controlled trial
Source: BMC Cancer. 2016 Apr 21;16:283. doi: 10.1186/s12885-016-2317-y (PMC4839098; doi:10.1186/s12885-016-2317-y)
Supplement: Additional file 10: — IRR for BC per 10 g alcohol/day for combinations PPARGC1A Gly482Ser and CYP19A1 genotypes. (DOCX 29 kb) [file 12885_2016_2317_MOESM10_ESM.docx]

**Additional file 10: IRR for BC per 10 g alcohol/day for combinations *PPARGC1A* Gly^482^Ser and *CYP19A1* genotypes**

| Genotype | PPARGC1A Gly^482^Ser | | PPARGC1A Gly^482^Ser | | PPARGC1A Gly^482^Ser | | P-value^c^ |
| --- | --- | --- | --- | --- | --- | --- | --- |
|  | Gly-carriers  n_cases_/ n_controls_  (n=650) | Ser/Ser  n_case_/ n_controls_  (n=650) | Gly-carriers  IRR (95% CI)^a^ | Ser/Ser  IRR (95% CI) ^a^ | Gly-carriers  IRR (95% CI)^b^ | Ser/Ser  IRR (95% CI)^b^ |  |
| rs10519297  AA  AG+GG | 139/136  437/427 | 20/26  54/61 | 1.16 (0.97-1.38)  1.17 (1.07-1.29) | 0.97 (0.59-1.59)  1.12 (0.89-1.42) | 1.13 (0.94-1.36)  1.16 (1.05-1.27) | 0.99 (0.59-1.63)  1.10 (0.87-1.40) | 0.92 |
| rs749292  GG  AG+AA | 185/172  391/391 | 23/21  41/66 | 1.27 (1.09-1.49)  1.13 (1.03-1.25) | 1.05 (0.77-1.44)  1.11 (0.83-1.47) | 1.27 (1.08-1.49)  1.11 (1.00-1.22) | 1.08 (0.79-1.49)  1.06 (0.79-1.42) | 0.50 |
| rs1062033  CC  CG+GG | 173/157  403/406 | 23/20  41/67 | 1.25 (1.06-1.47)  1.14 (1.04-1.26) | 1.16 (0.81-1.64)  1.04 (0.79-1.38) | 1.24 (1.05-1.46)  1.13 (1.02-1.24) | 1.19 (0.84-1.69)  1.00 (0.75-1.33) | 0.59 |
| rs10046  AA  AG+GG | 152/152  424/411 | 19/24  55/63 | 1.18 (0.99-1.40)  1.17 (1.06-1.28) | 0.99 (0.60-1.64)  1.12 (0.88-1.41) | 1.15 (0.97-1.38)  1.15 (1.05-1.27) | 1.00 (0.60-1.67)  1.10 (0.87-1.39) | 0.94 |
| rs4646  CC  CA+AA | 311/305  265/258 | 43/44  31/43 | 1.15 (1.03-1.28)  1.20 (1.05-1.35) | 1.06 (0.76-1.47)  1.12 (0.85-1.47) | 1.13 (1.01-1.26)  1.18 (1.04-1.34) | 1.01 (0.72-1.42)  1.14 (0.86-1.50) | 0.84 |
| rs6493487  AA  GA+GG | 342/345  234/218 | 42/59  32/28 | 1.14 (1.02-1.28)  1.19 (1.05-1.36) | 1.11 (0.86-1.44)  1.08 (0.74-1.57) | 1.12 (1.00-1.26)  1.18 (1.04-1.34) | 1.12 (0.87-1.45)  1.03 (0.70-1.52) | 0.89 |
| rs2008691  AA  GA+GG | 403/381  173/182 | 49/61  25/26 | 1.16 (1.05-1.28)  1.19 (1.02-1.40) | 0.97 (0.73-1.28)  1.42 (0.91-2.21) | 1.14 (1.04-1.26)  1.17 (1.01-1.37) | 0.94 (0.71-1.25)  1.40 (0.91-2.13) | 0.43 |
| rs3751591  TT+TC  CC | 551/552  25/11 | 74/85  -/2 | 1.16 (1.07-1.26)  3.85 (1.02-14.58) | 1.12 (0.90-1.39)  - | 1.14 (1.05-1.24)  3.70 (0.99-13.85) | 1.11 (0.89-1.38)  - | 0.37 |
| rs2445762  TT  TC+CC | 303/301  273/262 | 39/46  35/41 | 1.13 (1.01-1.26)  1.22 (1.08-1.38) | 1.21 (0.91-1.60)  0.95 (0.68-1.33) | 1.10 (0.98-1.23)  1.21 (1.07-1.37) | 1.19 (0.90-1.58)  0.94 (0.67-1.32) | 0.42 |
| rs11070844  CC  TC+TT | 463/451  113/113 | 57/74  17/13 | 1.17 (1.07-1.29)  1.15 (0.96-1.38) | 1.13 (0.86-1.49)  0.97 (0.69-1.36) | 1.16 (1.05-1.27)  1.14 (0.95-1.37) | 1.10 (0.83-1.46)  0.98 (0.70-1.38) | 0.83 |

^a^Crude.

^b^Adjusted for parity (parous/nulliparous, number of births, age at first birth), length of school education (low, medium, high), duration of HRT use (years) and body mass index (kg/m2).

^c^P-value for comparison of the adjusted risk estimates.
