# Supplementary material for: Alcohol-related breast cancer in postmenopausal women – effect of CYP19A1, PPARG and PPARGC1A polymorphisms on female sex-hormone levels and interaction with alcohol consumption and NSAID usage in a nested case-control study and a randomised controlled trial
Source: BMC Cancer. 2016 Apr 21;16:283. doi: 10.1186/s12885-016-2317-y (PMC4839098; doi:10.1186/s12885-016-2317-y)
Supplement: Additional file 11: — IRR for BC per 10 g alcohol/day for combinations of PPARGC1A Thr612Met and CYP19A1 genotypes. (DOCX 28 kb) [file 12885_2016_2317_MOESM11_ESM.docx]

**Additional file 11: IRR for BC per 10 g alcohol/day for combinations of *PPARGC1A* Thr^612^Met and *CYP19A1* genotypes**

| Genotype | PPARGC1A Thr^612^Met | | PPARGC1A Thr^612^Met | | PPARGC1A Thr^612^Met | | P-value^c^ |
| --- | --- | --- | --- | --- | --- | --- | --- |
|  | Thr/Thr  n_cases_/ n_controls_  (n=650) | Met-carriers  n_case_/ n_controls_  (n=650) | Thr/Thr  IRR (95% CI)^a^ | Met-carriers  IRR (95% CI) ^a^ | Thr/Thr  IRR (95% CI)^b^ | Met-carriers  IRR (95% CI)^b^ |  |
| rs10519297  AA  AG+GG | 144/154  445/440 | 15/8  56/48 | 1.11 (0.92-1.33)  1.16 (1.06-1.28) | 1.24 (0.75-2.06)  1.15 (0.88-1.52) | 1.10 (0.91-1.32)  1.15 (1.05-1.26) | 1.22 (0.72-2.06)  1.11 (0.85-1.47) | 0.96 |
| rs749292  GG  AG+AA | 190/174  399/424 | 18/22  43/34 | 1.23 (1.06-1.43)  1.12 (1.01-1.24) | 1.24 (0.73-2.11)  1.13 (0.86-1.48) | 1.23 (1.06-1.44)  1.10 (0.99-1.22) | 1.21 (0.71-2.06)  1.09 (0.83-1.43) | 0.63 |
| rs1062033  CC  CG+GG | 182/156  407/438 | 14/20  47/36 | 1.22 (1.05-1.43)  1.13 (1.02-1.24) | 1.40 (0.79-2.47)  1.10 (0.85-1.43) | 1.22 (1.04-1.43)  1.11 (1.01-1.23) | 1.36 (0.77-2.40)  1.06 (0.81-1.38) | 0.64 |
| rs10046  AA  AG+GG | 155/167  434/427 | 16/9  45/47 | 1.13 (0.95-1.35)  1.16 (1.05-1.27) | 1.31 (0.78-2.21)  1.14 (0.87-1.49) | 1.12 (0.93-1.33)  1.14 (1.04-1.26) | 1.28 (0.74-2.20)  1.10 (0.84-1.44) | 0.96 |
| rs4646  CC  CA+AA | 315/328  274/266 | 39/21  22/35 | 1.11 (0.99-1.25)  1.19 (1.06-1.34) | 1.25 (0.87-1.79)  0.91 (0.58-1.42) | 1.09 (0.97-1.23)  1.19 (1.05-1.34) | 1.21 (0.84-1.73)  0.86 (0.55-1.36) | 0.49 |
| rs6493487  AA  GA+GG | 348/375  241/219 | 36/30  25/26 | 1.13 (1.02-1.26)  1.18 (1.03-1.34) | 1.16 (0.85-1.58)  1.24 (0.87-1.77) | 1.12 (1.00-1.25)  1.16 (1.02-1.33) | 1.11 (0.80-1.52)  1.22 (0.85-1.73) | 0.95 |
| rs2008691  AA  GA+GG | 406/405  183/189 | 46/37  15/19 | 1.11 (1.01-1.23)  1.24 (1.07-1.45) | 1.24 (0.94-1.64)  0.86 (0.45-1.64) | 1.10 (1.00-1.21)  1.22 (1.05-1.42) | 1.20 (0.91-1.59)  0.80 (0.41-1.58) | 0.46 |
| rs3751591  TT+TC  CC | 565/584  24/10 | 60/53  1/3 | 1.15 (1.06-1.24)  1.46 (0.76-2.79) | 1.17 (0.92-1.48)  - | 1.13 (1.04-1.23)  1.50 (0.79-2.85) | 1.13 (0.90-1.44)  - | 0.86 |
| rs2445762  TT  TC+CC | 305/317  284/277 | 33/29  28/27 | 1.13 (1.01-1.26)  1.18 (1.05-1.32) | 1.15 (0.87-1.53)  1.24 (0.81-1.91) | 1.11 (0.99-1.24)  1.17 (1.04-1.32) | 1.10 (0.83-1.47)  1.21 (0.78-1.86) | 0.90 |
| rs11070844  CC  TC+TT | 473/478  116/116 | 46/47  15/9 | 1.18 (1.08-1.29)  1.07 (0.90-1.26) | 1.07 (0.82-1.41)  1.62 (0.84-3.10) | 1.16 (1.06-1.28)  1.07 (0.90-1.26) | 1.03 (0.78-1.36)  1.51 (0.80-2.84) | 0.55 |

^a^Crude.

^b^Adjusted for parity (parous/nulliparous, number of births, age at first birth), length of school education (low, medium, high), duration of HRT use (years) and body mass index (kg/m2) at baseline.

^c^P-value for comparison of the adjusted risk estimates.
