# Supplementary material for: Alcohol-related breast cancer in postmenopausal women – effect of CYP19A1, PPARG and PPARGC1A polymorphisms on female sex-hormone levels and interaction with alcohol consumption and NSAID usage in a nested case-control study and a randomised controlled trial
Source: BMC Cancer. 2016 Apr 21;16:283. doi: 10.1186/s12885-016-2317-y (PMC4839098; doi:10.1186/s12885-016-2317-y)
Supplement: Additional file 12: — IRR for BC per 10 g alcohol/day in relation to combinations of NSAID use and CYP19A1 polymorphisms. (DOCX 29 kb) [file 12885_2016_2317_MOESM12_ESM.docx]

**Additional file 12. IRR for BC per 10 g alcohol/day in relation to combinations of NSAID use and *CYP19A1* polymorphisms.**

| Genotype | No  n_cases_/  n_controls_  (n=640) | Yes  n_cases_/  n_controls_  (n=640) | No  IRR (95% CI)^a^ | Yes  IRR (95% CI)^a^ | No  IRR (95% CI)^b^ | Yes  IRR (95% CI)^b^ | *P*-value^c^ |
| --- | --- | --- | --- | --- | --- | --- | --- |
| rs10519297  AA  AG+GG | 81/104  286/315 | 76/55  197/166 | 1.16 (0.92-1.45)  1.12 (1.00-1.26) | 1.14 (0.89-1.46)  1.18 (1.04-1.35) | 1.13 (0.89-1.43)  1.10 (0.98-1.24) | 1.14 (0.88-1.47)  1.17 (1.02-1.33) | 0.94 |
| rs749292  GG  AG+AA | 119/128  248/291 | 86/63  187/158 | 1.14 (0.95-1.37)  1.13 (0.99-1.28) | 1.35 (1.06-1.69)  1.11 (0.97-1.28) | 1.14 (0.95-1.38)  1.10 (0.96-1.25) | 1.33 (1.06-1.68)  1.09 (0.95-1.26) | 0.49 |
| rs1062033  CC  CG+GG | 111/115  256/304 | 82/58  191/163 | 1.16 (0.95-1.41)  1.12 (0.99-1.27) | 1.30 (1.03-1.64)  1.13 (0.99-1.29) | 1.17 (0.96-1.43)  1.09 (0.96-1.24) | 1.28 (1.02-1.61)  1.12 (0.97-1.28) | 0.67 |
| rs10046  AA  AG+GG | 87/113  280/306 | 81/59  192/162 | 1.17 (0.94-1.47)  1.12 (0.99-1.25) | 1.18 (0.93-1.50)  1.17 (1.03-1.34) | 1.14 (0.90-1.44)  1.10 (0.97-1.24) | 1.17 (0.91-1.50)  1.16 (1.01-1.32) | 0.93 |
| rs4646  CC  CA+AA | 193/219  174/200 | 157/122  116/99 | 1.12 (0.97-1.29)  1.14 (0.98-1.33) | 1.16 (0.99-1.36)  1.19 (1.00-1.43) | 1.09 (0.94-1.26)  1.13 (0.97-1.32) | 1.14 (0.98-1.34)  1.18 (0.99-1.41) | 0.91 |
| rs6493487  AA  GA+GG | 209/263  158/156 | 169/136  104/87 | 1.14 (0.99-1.31)  1.11 (0.94-1.30) | 1.14 (0.97-1.33)  1.23 (1.02-1.48) | 1.12 (0.98-1.29)  1.08 (0.91-1.28) | 1.12 (0.96-1.31)  1.21 (1.01-1.46) | 0.83 |
| rs2008691  AA  GA+GG | 253/282  114/137 | 192/153  81/68 | 1.11 (0.99-1.26)  1.18 (0.97-1.43) | 1.15 (1.00-1.31)  1.25 (1.00-1.56) | 1.08 (0.96-1.23)  1.17 (0.96-1.43) | 1.14 (0.99-1.31)  1.21 (0.97-1.50) | 0.81 |
| rs3751591  TT+TC  CC | 350/409  17/10 | 265/218  8/3 | 1.12 (1.00-1.24)  1.47 (0.76-2.82) | 1.17 (1.04-1.32)  32 (0.42-2483) | 1.09 (0.97-1.21)  1.60 (0.83-3.08) | 1.16 (1.03-1.31)  26.0 (0.32-2191) | 0.29 |
| rs2445762  TT  TC+CC | 192/217  175/192 | 142/122  131/99 | 1.16 (1.00-1.35)  1.10 (0.96-1.27) | 1.09 (0.95-1.26)  1.32 (1.09-1.61) | 1.13 (0.97-1.32)  1.09 (0.94-1.26) | 1.07 (0.93-1.23)  1.33 (1.09-1.62) | 0.35 |
| rs11070844  CC  TC+TT | 303/340  64/79 | 207/180  66/41 | 1.18 (1.06-1.33)  0.91 (0.71-1.16) | 1.14 (0.99-1.30)  1.32 (1.00-1.75) | 1.16 (1.03-1.31)  0.89 (0.69-1.16) | 1.12 (0.98-1.28)  1.31 (1.00-1.72) | 0.20 |

^a^Crude.

^b^Adjusted for parity (parous/nulliparous, number of births, age at first birth), length of school education (low, medium, high), duration of HRT use (years) and body mass index (kg/m^2^) at baseline.

^c^*P*-value for comparison of the adjusted risk estimates.
