# Supplementary material for: Alcohol-related breast cancer in postmenopausal women – effect of CYP19A1, PPARG and PPARGC1A polymorphisms on female sex-hormone levels and interaction with alcohol consumption and NSAID usage in a nested case-control study and a randomised controlled trial
Source: BMC Cancer. 2016 Apr 21;16:283. doi: 10.1186/s12885-016-2317-y (PMC4839098; doi:10.1186/s12885-016-2317-y)
Supplement: Additional file 13: — Hormones, SHBG and ethanol measurements after acute ingestion of alcohol from the RCT study. (DOCX 19 kb) [file 12885_2016_2317_MOESM13_ESM.docx]

**Additional file 13. Hormones, SHBG and ethanol measurements after acute ingestion of alcohol from the RCT study.**

|  | *PPARG* wild types (n=18) | | *PPARG* ^12^Ala carriers (n=7) | |
| --- | --- | --- | --- | --- |
|  | Alcohol+  placebo | Alcohol+  Ibuprofen | Alcohol+  placebo | Alcohol+  Ibuprofen |
| Estrone pmol/l |  |  |  |  |
| 0 min | 26.25±12.94 | 27.50±16.53 | 14.60±7.31 | 31.50±2.50 |
| 30 min | 16.50±13.10 | 15.23±10.17 | 10.00±10.00 | 19.00±15.58 |
| 60 min | 15.36±10.41 | 11.60±9.19 | 9.83±8.90 | 8.00±8.04 |
| 90 min | 13.20±12.69 | 9.00±9.18 | 13.67±9.88 | 13.50±9.54 |
| 1200 min | 24.56±17.71 | 30.45±19.61 | 18.40±11.81 | 32.00±32.47 |
| Estrone sulphate pmol/l |  |  |  |  |
| 0 min | 797.45±493.44 | 731.39±473.95 | 492.29±162.22 | 714.79±422.03 |
| 30 min | 575.78±285.18 | 714.56±519.96 | 426.64±105.34 | 699.14±415.42 |
| 60 min | 578.29±473.07 | 627.67±452.80 | 413.36±110.07 | 640.21±395.21 |
| 90 min | 750.38±527.00 | 659.56±496.70 | 428.07±124.82 | 652.29±389.76 |
| 1200 min | 626.89±375.54 | 698.11±505.23 | 468.29±149.23 | 871.14±764.29 |
| SHBG nmol/l |  |  |  |  |
| 0 min | 64.96±26.99 | 65.01±22.27 | 69.23±16.00 | 66.58±13.90 |
| 30 min | 61.62±21.74 | 67.75±25.31 | 71.18±17.00 | 68.26±13.80 |
| 60 min | 62.41±23.93 | 61.38±18.45 | 65.18±12.54 | 66.32±12.08 |
| 90 min | 59.90±23.59 | 62.82±21.64 | 69.51±16.20 | 59.67±13.12 |
| 1200 min | 65.39±30.15 | 64.58±22.25 | 70.58±15.71 | 65.51±22.00 |
| Ethanol g/l |  |  |  |  |
| 0 min | <0.00 | <0.00 | <0.00 | <0.00 |
| 30 min | 0.19±0.12 | 0.20±0.11 | 0.11±0.076 | 0.19±0.086 |
| 60 min | 0.32±0.094 | 0.34±0.088 | 0.29±0.036 | 0.33±0.048 |
| 90 min | 0.30±0.070 | 0.31±0.062 | 0.28±0.028 | 0.32±0.047 |
| 1200 min | <0.00 | <0.00 | <0.00 | <0.00 |

Blood concentrations of estrone, estrone sulphate, SHBG and ethanol before and after 30, 60, 90 and 1200 minutes of drinking, respectively. Values are presented as means ± SD.
